# Inflammatory blood-based biomarkers to aid in the assessment and prognostication of traumatic brain injury: a TRACK-TBI study

**DOI:** 10.1186/s12974-026-03891-3

**Published:** 2026-05-28

**Authors:** John K. Yue, Allen Y. Fu, Sonia Jain, Ava M. Puccio, Shawn R. Eagle, Frederick K. Korley, Thomas A. van Essen, Romit Samanta, Lucia M. Li, Christopher J. Roberts, David J. Caldwell, Mahmoud M. Elguindy, Mary J. Vassar, Patrick J. Belton, Shubhayu Bhattacharyay, Lindsay D. Nelson, Joye X. Tracey, Leila L. Etemad, Christine J. Gotthardt, Gabriela G. Satris, Maxwell B. Wang, Catherine Demos, George B. Sigal, Edilberto Amorim, Debbie Y. Madhok, Hannah L. Radabaugh, Adam R. Ferguson, Amy J. Markowitz, Claudia S. Robertson, Alex B. Valadka, Pratik Mukherjee, Esther L. Yuh, Michael A. McCrea, H. E. Hinson, Andrea L. C. Schneider, Xiaoying Sun, David O. Okonkwo, Firas H. Kobeissy, Geoffrey T. Manley, Ramon Diaz-Arrastia, Kevin K. W. Wang, TRACK-TBI Investigators, Gretchen M. Brophy, Randall M. Chesnut, Ann-Christine Duhaime, Brian Fabian, Brandon Foreman, Joseph T. Giacino, Taron Gorham, Ramesh Grandhi, J. Russell Huie, Ruchira M. Jha, Vijay Krishnamoorthy, Hester F. Lingsma, Christine L. Mac Donald, Rebekah Mannix, Diego Martell, David K. Menon, Randall Merchant, Caroline Neely, Laura B. Ngwenya, Nikhil Padmanabhan, Kathryn S. Park, Richard B. Rodgers, David M. Schnyer, Murray B. Stein, Sabrina R. Taylor, Nancy R. Temkin, Theodore T. Tran, Jacob N. Wohlstadter, Ross D. Zafonte

**Affiliations:** 1 Department of Neurological Surgery, University of California, 2540 23Rd Street, Room 5204, San Francisco, CA 94110 USA; 2 Brain and Spinal Injury Center, Zuckerberg San Francisco General Hospital, San Francisco, CA USA; 3 Weill Institute for Neurosciences, University of California, 2540 23Rd Street, Room 5204, San Francisco, CA 94110 USA; 4 Philip R. Lee Institute for Health Policy Studies, University of California, 2540 23Rd Street, Room 5204, San Francisco, CA 94110 USA; 5 New York University Grossman School of Medicine, New York, NY USA; 6 Biostatistics Research Center, Herbert Wertheim School of Public Health and Longevity Science, University of California San Diego, San Diego, CA USA; 7 Department of Neurological Surgery, University of Pittsburgh Medical Center, Pittsburgh, PA USA; 8 Department of Emergency Medicine, University of Michigan, Ann Arbor, MI USA; 9 University Neurosurgical Center Holland, Leiden University Medical Center, Haaglanden Medical Center, HAGA, Leiden, The Hague, the Netherlands; 10 Department of Surgery, Division of Neurosurgery, QEII Health Sciences Center and Dalhousie University, Halifax, NS Canada; 11 Department of Medicine, University of Cambridge, Cambridge, UK; 12 Department of Medicine, Imperial College London, London, UK; 13 Department of Anesthesia, Medical College of Wisconsin, Milwaukee, WI USA; 14 Department of Neurological Surgery, University of Wisconsin-Madison, Madison, WI USA; 15 Harvard Medical School, Boston, MA USA; 16 Department of Neurological Surgery, Medical College of Wisconsin, Milwaukee, WI USA; 17 Meso Scale Diagnostics, LLC, Rockville, MD USA; 18 Department of Neurology, University of California, San Francisco, CA USA; 19 Department of Emergency Medicine, University of California, San Francisco, CA USA; 20 Department of Neurological Surgery, Baylor College of Medicine, Houston, TX USA; 21 Department of Neurological Surgery, University of Texas Southwestern Medical Center, Dallas, TX USA; 22 Department of Radiology and Biomedical Imaging, University of California, San Francisco, CA USA; 23 Department of Neurology, University of Pennsylvania, Philadelphia, PA USA; 24 Department of Biostatistics, Epidemiology, and Informatics, University of Pennsylvania, Philadelphia, PA USA; 25 Department of Neurobiology, Morehouse School of Medicine, Atlanta, GA USA

**Keywords:** Biomarkers, Cytokines, Diagnostic tests, Inflammation, Neuroinflammation, Prognostic factors, Recovery of function, Traumatic brain injury

## Abstract

**Background:**

Inflammatory proteins detectable in blood reflect pathoanatomic injury patterns after traumatic brain injury (TBI). Identifying biomarkers of secondary neurologic and systemic injury may improve detection of patients at risk for clinical decline and chronic disability. This study examined the utility of acute and subacute inflammatory biomarkers to differentiate TBI diagnosis and severity, and predict 6-month outcomes.

**Methods:**

The Transforming Research and Clinical Knowledge in Traumatic Brain Injury (TRACK-TBI) Study prospectively enrolled TBI patients presenting to 18 United States trauma centers with head computed tomography (CT) on day 1 (D1; < 24-h post-injury). Data were extracted from TRACK-TBI subjects with D1 and 2-week (W2) plasma samples and 6-month functional outcomes, yielding 369 TBI subjects, 100 orthopedic trauma controls (OC), and 69 healthy controls. Twenty-seven inflammatory biomarkers were analyzed (MesoScale Diagnostics). TBI severity was defined using Glasgow Coma Scale (GCS; 3–12/13–15) and radiographic intracranial injury (CT-positive/CT-negative). Multivariable logistic regressions examined biomarkers as predictors of 6-month unfavorable outcomes (Glasgow Outcome Scale-Extended = 1–4 (death/severe-disability)), and adjusted for clinico-demographic factors and multiple comparisons. Adjusted odds ratios (AOR [95% confidence interval]) per log_2_-unit increase in biomarker level were reported.

**Results:**

Ten biomarkers (c-reactive protein (CRP), serum amyloid A (SAA), interleukin (IL)-1ꞵ, IL-2, IL-4, IL-6, IL-10, IL-15, IL-17A, tumor necrosis factor (TNF)-α) differed significantly between GCS 3–12 vs. 13–15 TBI, CT-positive vs. CT-negative TBI, and GCS 3–12 TBI vs. OC, at both D1 and W2 (*p* < 0.001). IL-6, CRP, and SAA showed good discrimination of clinical TBI severity (D1/W2 area under-the-curve (AUC): 0.87/0.87, 0.82/0.88, 0.80/0.85, respectively), and moderate-to-good discrimination of radiographic TBI severity (D1/W2 AUC: 0.81/0.82, 0.78/0.83, 0.77/0.79, respectively). Five W2 biomarkers emerged as multivariable predictors of 6-month unfavorable outcomes (IL-15: AOR = 2.26 [1.14–4.49]; SAA: AOR = 1.91 [1.37–2.67]; IL-6: AOR = 1.80 [1.25–2.61]; IL-17A: AOR = 1.72 [1.24–2.39]; CRP: AOR = 1.40 [1.06–1.85]).

**Conclusions:**

Ten circulating inflammatory proteins were associated with TBI diagnosis and severity at D1 and W2. Of these, five biomarkers expressed subacute (W2) levels predictive of 6-month death/severe-disability, underscoring their potential for validation as a novel biomarker class and integration into TBI prognostic models. Distillation of pro- and anti-inflammatory biomarker cascades in TBI could facilitate precision medicine approaches for risk stratification and therapeutic modulation.

**Supplementary Information:**

The online version contains supplementary material available at 10.1186/s12974-026-03891-3.

## Background

Traumatic brain injury (TBI) is a leading cause of disability worldwide, affecting 50–60 million people each year [1]. Precision medicine approaches to predicting outcomes after TBI require understanding the molecular mechanisms associated with the trajectory of injury progression and tissue repair [2]. Blood-based biomarkers show significant promise as objective, meaningful indicators of TBI severity and prognosis. Proteomic biomarkers of structural central nervous system (CNS)-specific injury that are detectable in the systemic circulation have undergone considerable validation; S100 calcium-binding protein B (S100B) has been included as part of Scandinavian guidelines for severe TBI assessment since the early 2000 s [3–5], and in 2023 the United States of America (USA) Food and Drug Administration (FDA) qualified glial fibrillary acidic protein (GFAP) and ubiquitin c-terminal hydrolase-L1 (UCH-L1) to aid in the clinical diagnosis of acute TBI in certain subpopulations [6–8]. As evidence for expanding their clinical contexts of use continue to grow, inflammatory biomarkers have demonstrated growing promise for detecting secondary and evolving brain injuries as well as their sequelae.

TBI induces complex systemic and neuroinflammatory cascades including reactive astrogliosis, immune cell recruitment, and modulation of cytokines that activate systems of cellular repair, apoptosis, and secondary injury [9–13]. Greater understanding of TBI-related inflammatory cascades may create new opportunities to improve diagnosis and delineate therapeutic targets to prevent or temporize the propagation of ongoing CNS and systemic injuries and may provide a causal link to chronic neurodegeneration [10]. Even after mild TBI, inflammatory biomarkers have been associated with impairment of memory, cognition, and mental health [14]. In a previous study from our group of 160 prospectively enrolled acute TBI patients from the Transforming Research and Clinical Knowledge in Traumatic Brain Injury (TRACK-TBI) Pilot Study conducted across 3 USA Level 1 trauma centers between 2010–2012, a profile of plasma inflammatory biomarkers drawn within 24 h post-injury showed promising discriminatory ability for clinical and radiographic TBI severity by Glasgow Coma Scale score (GCS; 3–12 vs. 13–15), CT findings of intracranial injury, and unfavorable 6-month functional outcome, of which interleukin (IL)−15 and serum amyloid A (SAA) had the highest areas under the receiver operating characteristic (ROC) curve (AUC; > 0.7) across these indications [15, 16]. However, this study was limited by a relatively small sample size, a need for further demographic representation, a limited number of participants with severe TBI, and measurement of biomarkers only within 24 h of injury. While the acute inflammatory response reflects immediate injury severity, subacute inflammation may serve as an index of maladaptive recovery trajectories [17]. We therefore sought to interrogate the unique prognostic characteristics of inflammatory biomarkers at different time points post injury.

There remains a need for large-scale, high-quality studies from contemporary TBI populations to confirm the diagnostic and prognostic utility of candidate inflammatory markers. The current study aimed to examine the performance of 27 acute and subacute proteomic inflammatory biomarkers for TBI severity detection and 6-month outcome prediction using prospectively collected data from the TRACK-TBI cohort study conducted across 18 USA Level 1 trauma centers between 2014–2019. Specifically, plasma biomarker levels on day 1 (D1, within 24 h) and at 2-weeks (W2) post-injury were assessed for their abilities to differentiate clinical and radiographic TBI severity, and predict 6-month unfavorable functional outcomes.

## Methods

### TRACK-TBI study overview

The TRACK-TBI Study (ClinicalTrials.gov #NCT02119182) was an observational cohort study which prospectively enrolled patients through convenience sampling from March 2, 2014 to June 22, 2019 across 18 USA Level 1 trauma centers. Enrollment and data collection methodologies adhered to the National Institute of Neurological Disorders and Stroke (NINDS) TBI Common Data Elements (CDEs) and have been previously described [8, 18, 19]. Inclusion criteria for the TRACK-TBI Study were presentation to the emergency department (ED) of a participating site with one or more clinical symptoms or signs that met the American Congress of Rehabilitation Medicine definition for TBI (loss of consciousness, post-traumatic amnesia, alteration of consciousness, or neurologic deficit) and receiving a clinically indicated head computed tomography (CT) scan within 24 h of blunt external force head trauma. Exclusion criteria for the TRACK-TBI Study included pregnancy, prisoners or in custody of law enforcement, psychiatric hold, penetrating TBI, significant polytrauma that could interfere with validity of outcome assessments as assessed by the site principal investigator, major/debilitating medical (e.g. end-stage malignancy, refractory substance abuse, or transmittable disease that would preclude informed consent), neurological (e.g. cerebrovascular accident, central nervous system malignancy, cognitive impairment), or mental health conditions (e.g. schizophrenia) that could interfere with validity of outcome assessments as assessed by the site principal investigator, non-English and non-Spanish primary language, and ongoing participation in an interventional trial (drug, device, behavioral) [8, 18, 19].

Orthopedic control (OC) subjects presented with acute traumatic injuries to the limbs, chest/thorax, abdomen and/or pelvis with an Abbreviated Injury Scale (AIS) score of 1–3 to these body systems, without acute injury to the head or neck, and without loss or alteration of consciousness, post-traumatic amnesia, or clinical signs of head injury. Healthy controls (HC) were recruited from friends/relatives of TRACK-TBI subjects or through public advertisement at participating sites, and did not have history of TBI, concussion, or traumatic injury within 12 months prior to enrollment [6].

### Ethical approval and informed consent

The TRACK-TBI Study was approved by the institutional review board (IRB) at each of the 18 participating Level 1 trauma centers. Human subjects research conducted during the TRACK-TBI Study followed the principles of the Declaration of Helsinki. Written consent was obtained from each subject or their legally authorized representatives before study enrollment. The Galveston Orientation and Amnesia Test was used to determine competency to give informed consent, with a passing score of 76–100 [6]. Subjects who did not achieve a passing score on the Galveston Orientation and Amnesia Test or were unable to provide informed consent due to the severity of their injuries at the time of enrollment were enrolled by surrogate consent from their legally authorized representative, and competency screening was repeated with the subject prior to each follow-up visit to determine suitability for written informed consent. The current study is a secondary analysis of previously collected TRACK-TBI data and did not require separate IRB approval.

### Analytic cohort selection

**Fig. 1:**
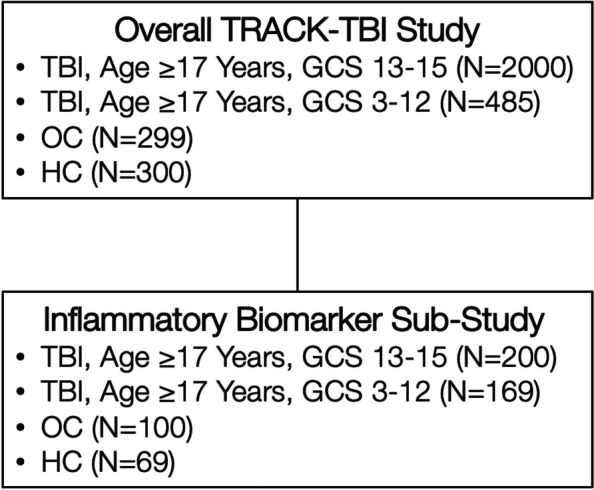
Study Flow Diagram of Included Subjects. A subset of adult subjects with traumatic brain injury (TBI), orthopedic controls (OC), and healthy controls (HC) in the Transforming Research and Clinical Knowledge in Traumatic Brain Injury (TRACK-TBI) study were selected for inclusion in the current study. The sample size was determined a priori* due* to cost considerations. Data were extracted from TRACK-TBI subjects with adequate D1 and W2 blood samples for inflammatory biomarker assays and complete 6-month outcome assessments. Subjects were selected at random from the TRACK-TBI study cohort under the supervision of the TRACK-TBI Biostatistical Core. HCs only received biomarker measurements at D1. D1 = day 1; GCS = Glasgow Coma Scale; W2 = 2-week

A priority subset of data from TRACK-TBI subjects aged ≥ 17 years at time of injury was selected for inflammatory biomarker analysis. An a priori target of approximately 200 subjects within each of the GCS 3–12 and GCS 13–15 TBI groups, with D1 and W2 blood samples and completed 6-month outcomes assessments, was selected for analysis due to cost considerations for the biomarker assays. All subjects included in the current analysis contributed both D1 and W2 blood samples for biomarker analyses. Subjects for the GCS 3–12 and GCS 13–15 TBI groups were selected at random under the supervision of the TRACK-TBI Biostatistical Core, and resulted in sample sizes of 169 and 200 respectively. Blood samples from 100 OC and 69 HC subjects were analyzed for the current study. The Consolidated Standards of Reporting Trials (CONSORT) flow diagram for included study subjects is shown in Fig. 1.

### Demographic and clinical variables

Subjects were assessed at ED admission and throughout their hospitalization through in-person assessment and medical record review. Sociodemographic, clinical, and injury history variables were collected and recorded in accordance with the NINDS TBI Clinical CDEs by centrally trained research personnel in the TRACK-TBI Study [8, 18, 19]. Clinical and neurological assessments, including the GCS score and its components, were performed by the clinicians treating the patient as part of clinical care, and abstracted from the patient’s medical record by TRACK-TBI Study research personnel.

### Six-month outcome assessment

Functional outcomes of patients with TBI were assessed via the ordinal GOSE measure through structured interviews by trained research personnel [20], ranging from death (GOSE 1) to recovery to baseline function (GOSE 8). The GOSE assesses consciousness, function in and outside the home, ability to work/study, participation in social and leisure activities, family and friendships, and symptoms [21]. The primary endpoint of the current study was prediction of unfavorable vs. favorable 6-month outcome, and GOSE scores were dichotomized as unfavorable (GOSE 1–4), indicating death, vegetative state, or severe disability, or favorable (GOSE 5–8), indicating moderate disability or good recovery [21]. Additionally, prediction of incomplete vs. complete 6-month functional recovery was examined, using GOSE scores dichotomized as 1–7 (incomplete recovery) vs. 8 (complete recovery).

### Biomarker collection and measurement

Peripheral venipuncture was used to draw blood samples from TRACK-TBI subjects on D1 and W2. Blood samples were time-stamped and processed in accordance with the NINDS TBI Biospecimens and Biomarkers CDEs [8, 18, 19]. Specifically, blood samples were collected into K2EDTA (dipotassium ethylenediaminetetraacetic acid) vacutainer tubes, placed on ice for 5 to 10 min, then centrifuged at 4000 revolutions per minute for 5 to 7 min. Plasma aliquots of 500 µl were prepared for each subject, frozen at −80℃ in non-frost-free freezers, and batch shipped in temperature-controlled overnight express freight containers to the TRACK-TBI Biospecimens Repository at the University of Pittsburgh Medical Center (Pittsburgh, Pennsylvania, USA) [22].

Biomarker assays were analyzed using the Meso Scale Discovery (MSD) V-Plex Panels: Proinflammatory Panel 1 (Catalog #K15049D-1), Cytokine Panel 1 (#K15050D-1), Chemokine Panel 1 (#K15047D-1), and Vascular Injury Panel 2 (#K15198D-1) (Meso Scale Diagnostics, LLC, Rockville, Maryland, USA). We report results for the following 27 neuroinflammatory biomarkers: CRP, eotaxin, eotaxin-3, interferon (IFN)-γ, interleukin (IL)−1α, IL-1ꞵ, IL-2, IL-4, IL-5, IL-6, IL-7, IL-10, IL-12/23 p40 protein (IL-12/23p40), IL-12 p70 protein (IL-12p70), IL-15, IL-16, IL-17A, interferon gamma-induced protein 10 (IP-10), monocyte chemoattractant protein (MCP)−1, MCP-4, macrophage-derived chemokine (MDC), macrophage inflammatory protein (MIP)−1α, MIP-1ꞵ, serum amyloid A (SAA), thymus- and activation-regulated chemokine (TARC), tumor necrosis factor (TNF)-α, and TNF-ꞵ. Other non-inflammatory biomarkers from these panels were considered outside of the scope of this work [16].^.^

Biomarker assay characteristics are shown in Supplementary Table S1. Biomarker sample values below their lower limit of quantification (LLOQ) or above their upper limit of quantification (ULOQ) were treated as their respective LLOQ or ULOQ values for study analyses. Biomarkers with > 10% of values below LLOQ (IL-1ꞵ, IL-4, IL-5, TNF-ꞵ) or above ULOQ (SAA, IL-6) at each timepoint were reported: IL-1ꞵ (< LLOQ, TBI D1: 19%, TBI W2: 48%, OC D1: 52%, OC W2: 67%, HC: 91%), IL-4 (< LLOQ, TBI D1: 22%, TBI W2: 38%, OC D1: 43%, OC W2: 44%, HC: 63%), IL-5 (< LLOQ, TBI D1: 48%, TBI W2: 34%, OC D1: 39%, OC W2: 36%, HC: 56%), TNF-ꞵ (< LLOQ, TBI D1: 66%, TBI W2: 56%, OC D1: 69%, OC W2: 51%, HC: 38%), SAA (> ULOQ, TBI D1: 30%, TBI W2: 15%, OC D1: 11%) IL-6 (> ULOQ, TBI D1: 46%, OC D1: 12%).

The structural CNS-injury biomarker GFAP was included as a covariate in multivariable regression models for outcome prediction. Plasma samples for D1 GFAP were processed identically using the same blood draw as the inflammatory biomarkers, and assays were performed with the MSD S-PLEX Neurology Panel 1 Kit (Catalog #K15639S).

### Neuroimaging coding

The deidentified initial head CT scan from time of injury for each TRACK-TBI subject was uploaded to a central imaging database at the Laboratory of Neuro Imaging (University of Southern California, Los Angeles, California, USA). A board-certified neuroradiologist independently reviewed each image blinded to other research data, and images were coded in accordance with the NINDS TBI Neuroimaging CDEs [23–25]. CTs were considered positive for acute traumatic intracranial injury (CT +) if evidence of intracerebral contusions, traumatic subarachnoid hemorrhage, subdural hematoma, epidural hematoma, axonal shear injury, intraventricular hemorrhage, midline shift, or downward herniation were present. Images were otherwise read as negative (CT-). The Marshall CT Classification Score [26], historically used to describe traumatic brain injury severity and predict outcomes, was determined by the TRACK-TBI neuroradiologist. The coded results were uploaded to the TRACK-TBI clinical database.

### Statistical analysis

Descriptive statistics were generated for the study cohort by TBI diagnostic and severity groups. Group comparisons used the Wilcoxon rank sum test for non-normally distributed continuous variables and Fisher’s exact test for the categorical variables. Biomarker values were log₂-transformed to reduce the influence of skewed distributions. Fold changes were calculated using the medians of log₂-transformed biomarker levels to ensure that group differences reflect multiplicative changes on a stabilized scale. Pairwise correlations among the biomarkers were assessed using Spearman’s rank correlation coefficient and visualized with a heatmap. A correlation coefficient ≥ 0.4 was considered moderate, ≥ 0.6 was considered strong, and ≥ 0.8 was considered very strong [27]. The discriminative ability of each biomarker for TBI diagnosis and severity was assessed using the area under the receiver-operating characteristic curve (AUC) with 95% confidence intervals (CI). AUC of 0.7–0.8 was considered adequate discrimination, and AUC of 0.8–0.9 was considered good discrimination [6]. For biomarker group comparisons, statistical significance was defined using a Bonferroni-adjusted threshold of *p* < 0.0018 (0.05/27 biomarkers).

The prognostic ability of each biomarker at D1 and W2 for 6-month functional outcomes (GOSE) was examined using multivariable logistic regression models. Separate models were fit for each biomarker and were adjusted for the same set of covariate predictors with known associations with TBI outcomes: age (continuous), sex (male vs. female), major extracranial injury (defined as AIS score ≥ 3 for any extracranial body system; yes vs. no), ED arrival GCS (3–12 vs. 13–15), psychiatric history (yes vs. no), Marshall CT scores (2, 3–4, 5–6 vs. 1) and D1 GFAP levels (continuous in log_2_ scale). Adjusted odds ratios (AOR) with 95% CIs were reported. For biomarkers, the odds ratio represents the change in odds per log_2_ unit increase. *P*-values were corrected for multiple comparisons where indicated using the Benjamini–Hochberg method.

Level and duration of inpatient care are indicators of clinical severity and complexity, and may influence both the inflammatory response and TBI outcomes. A sensitivity analysis was conducted examining the association between W2 biomarker levels and 6-month unfavorable outcomes to account for potential confounding from ongoing and/or prolonged hospitalization. We focused on 10 biomarkers that demonstrated consistent diagnostic performance across clinico-radiographic TBI classification categories (GCS 3–12 vs. 13–15, CT + vs. CT-, and TBI vs. OC comparisons at both D1 and W2). Hospitalization status at time of W2 blood draw (not hospitalized, hospitalized not in intensive care unit (ICU), hospitalized in ICU) was added as a covariate predictor to multivariable logistic regression models. AORs were reported for biomarkers, with *p*-values corrected for multiple comparisons using the Benjamini–Hochberg method.

Given prior evidence of sex-related differences in plasma interleukin levels at 7 days post-injury in certain TBI subcohorts [28], a separate exploratory analysis was performed to examine whether biomarker performance at W2 varied by sex. For the same set of 10 biomarkers that demonstrated consistent diagnostic performance, we added a biomarker-by-sex interaction term to multivariable logistic regression models for the primary outcome. As this was exploratory, *p*-values were not adjusted for multiple comparisons.

Statistical analyses were conducted using R version 4.4.1 (http://www.r-project.org).

## Results

### Baseline and injury characteristics

Three-hundred sixty-nine TBI subjects, 100 OCs and 69 HCs were included in the current analysis. Baseline characteristics of GCS 13–15 TBI, GCS 3–12 TBI, and OC subjects are shown in Table 1. In HC subjects, median age was 30 years [Q1-Q3: 24–40] and 71% were male. Other demographic information was not available for HCs. Median time from injury to blood sample collection was 2 h longer in GCS 3–12 subjects compared to GCS 13–15.

**Table 1:** Demographic and clinical characteristics of the analytic cohort

Variable	GCS 13–15 TBI N (%)	GCS 3–12 TBI N (%)	OC N (%)	*p*-value (GCS 13–15 TBI vs. GCS 3–12 TBI)	*p*-value (GCS 13–15 TBI vs. OC)	*p*-value (GCS 3–12 TBI vs. OC)
Age						
Median [Q1-Q3] (years)	39 [27–52]	35 [25–51]	37 [28–54]	0.088	0.951	0.143
Time to sample collection on D1						
Median [Q1-Q3] (hours)	15 [9–21]	17 [12–22]	14 [5–21]	0.012	0.126	0.001
Sex				0.006	0.378	0.001
Male	129 (65)	132 (78)	59 (59)			
Female	71 (36)	37 (22)	41 (41)			
Total	200 (100)	169 (100)	100 (100)			
Race				0.456	0.794	0.601
White	151 (76)	135 (80)	77 (79)			
Black	34 (17)	21 (13)	16 (16)			
Other	15 (8)	12 (7)	5 (5)			
Total	200 (100)	168 (100)	98 (100)			
Ethnicity				> 0.999	0.210	0.262
Non-Hispanic	166 (83)	139 (83)	75 (77)			
Hispanic	34 (17)	29 (17)	23 (23)			
Total	200 (100)	168 (100)	98 (100)			
Psychiatric History				0.385	0.488	0.139
No	150 (75)	134 (79)	71 (71)			
Yes	50 (25)	35 (21)	29 (29)			
Total	200 (100)	169 (100)	100 (100)			
Prior TBI				0.008	0.138	0.593
No	137 (73)	128 (85)	75 (82)			
Yes	51 (27)	23 (15)	17 (18)			
Total	188 (100)	151 (100)	92 (100)			
Injury Cause				0.335	< 0.001	< 0.001
Road traffic incident	130 (65)	101 (60)	35 (36)			
Incidental fall	46 (23)	37 (22)	37 (38)			
Violence/assault	11 (6)	10 (6)	1 (1)			
Other	13 (7)	20 (12)	24 (25)			
Total	200 (100)	168 (100)	97 (100)			
ED Disposition				< 0.001	< 0.001	< 0.001
ED Discharge	63 (32)	0 (0)	50 (50)			
Hospital Ward	85 (43)	1 (0.6)	45 (45)			
Intensive Care Unit	52 (26)	168 (99)	5 (5)			
Total	200 (100)	169 (100)	100 (100)			
Major Extracranial Injury				< 0.001	0.868	< 0.001
No	168 (84)	109 (64.5)	85 (85)			
Yes	32 (16)	60 (35.5)	15 (15)			
Total	200 (100)	169 (100)	100 (100)			
Loss of Consciousness				< 0.001		
No	32 (16)	2 (1)	-			
Yes	161 (81)	161 (95)	-			
Unknown	7 (4)	6 (4)	-			
Total	200 (100)	169 (100)	-			
Post-Traumatic Amnesia				< 0.001		
No	27 (14)	4 (2.4)	-			
Yes	157 (79)	109 (65)	-			
Unknown	16 (8)	56 (33)	-			
Total	200 (100)	169 (100)	-			
Intracranial Injury on CT				< 0.001		
CT-	134 (67)	14 (8)	-			
CT +	66 (33)	155 (92)	-			
Total	200 (100)	169 (100)	-			
Marshall CT Score				< 0.001		
1	134 (67)	14 (8)	-			
2	59 (30)	75 (45)	-			
3–4	3 (2)	15 (9)	-			
5–6	4 (2)	61 (37)	-			
Total	200 (100)	165 (100)	-			

Caption: Demographic and clinical characteristics of patients with moderate-to-severe TBI (GCS 3–12), mild TBI (GCS 13–15), and orthopedic controls. Major extracranial injury represented injury to a body system below the head and neck with an Abbreviated Injury Scale score ≥ 3. Parentheses represent percentages. CT = computed tomography; ED = emergency department; GCS = Glasgow Coma Scale; ICU = intensive care unit; OC = orthopedic trauma control; Q1-Q3 = quartile 1 to quartile 3; TBI = traumatic brain injury

### Inflammatory biomarker correlations in TBI, OC and HC groups

**Fig. 2:**
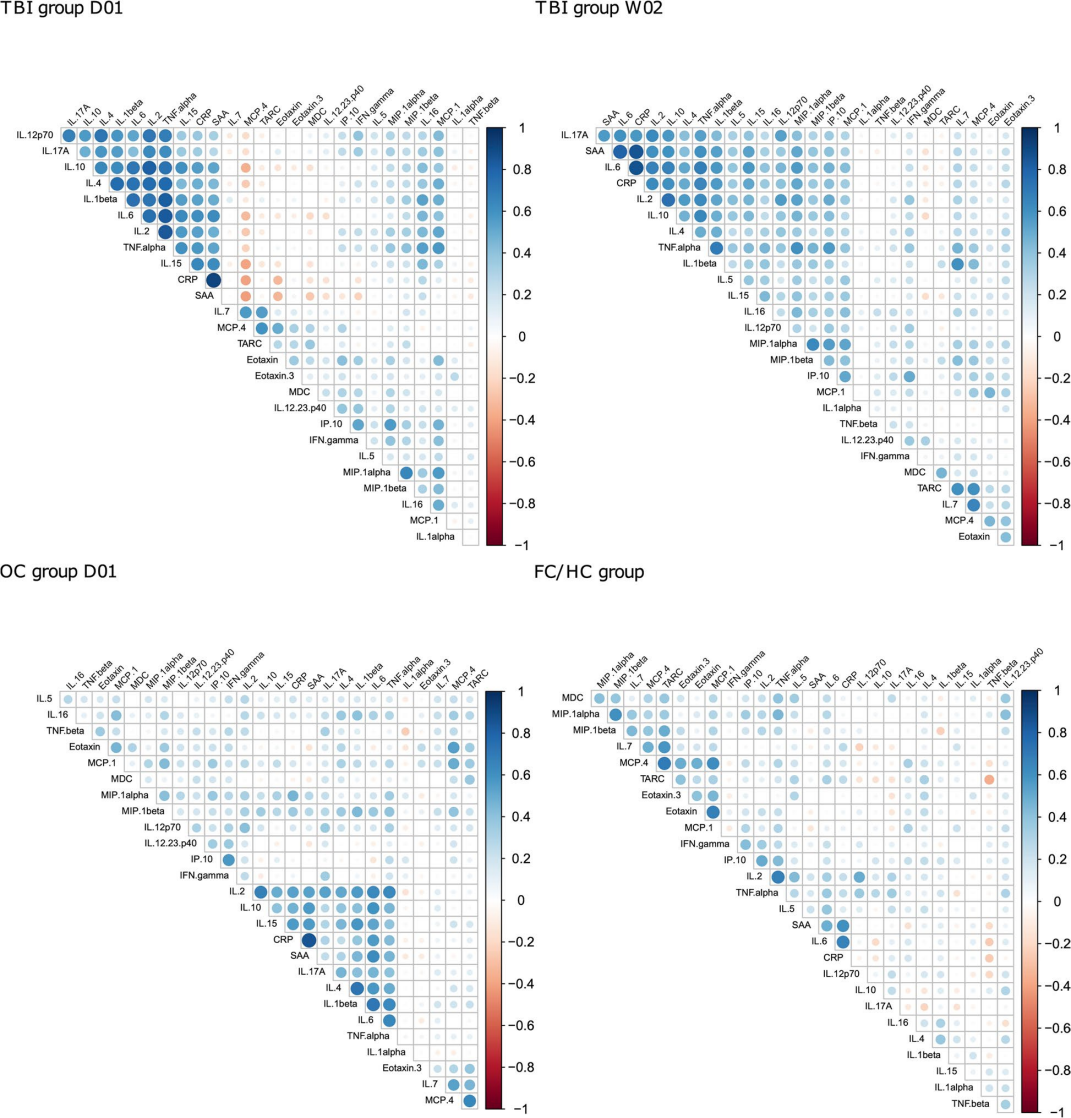
Spearman’s Correlations of Inflammatory Biomarkers in TBI Subjects and Controls. Spearman’s rank correlation matrices for inflammatory markers in TBI subjects at day 1 (D1), TBI subjects at 2-weeks (W2) post-injury, orthopedic controls, and healthy controls. Blue and red colors signify positive and negative correlations, respectively. CRP = c-reactive protein; D1 = day 1; FC/HC = friend control/healthy control; IFN = interferon; IL = interleukin; IP = interferon-gamma induced protein; MCP = monocyte chemoattractant protein; MDC = macrophage-derived chemokine; MIP = macrophage inflammatory protein; OC = orthopedic trauma control; SAA = serum amyloid A; TARC = thymus and activation regulated chemokine; TBI = traumatic brain injury; TNF = tumor necrosis factor; W2 = 2-week

Of D1 biomarkers in the TBI group, CRP and SAA were the most strongly correlated (ρ = 0.92). IL-1ꞵ, IL-2, IL-4, IL-6, IL-10, IL-12p70, IL-15, IL-17A, and TNF-α demonstrated strong positive correlations with each other. An isolated correlation was observed between MIP-1α and MIP-1ꞵ (ρ = 0.64) (Fig. 2). Of W2 biomarkers in the TBI group, CRP, IL-6, and SAA remained very strongly correlated, and IL-2, IL-6, IL-10, IL-17A, SAA, CRP, and TNF-ɑ showed strong positive correlations with each other (Fig. 2).

The OC group showed a very strong correlation between CRP and SAA (ρ = 0.86), and strong correlations among IL-1ꞵ, IL-2, IL-4, IL-6, IL-10, and TNF-ɑ. An isolated correlation was observed between MCP-4 and TARC (Fig. 2). The HC group showed strong correlations between CRP/SAA, CRP/IL-6, IL-2/TNF-α, MCP-4/TARC, MCP-1/MCP-4, MIP-1α/MIP-1ꞵ, and eotaxin/MCP-1 (Fig. 2).

Compared to the OC group, the TBI group showed unique D1 correlations between IL-12p70, IL-15, and IL-17A and an increased number of very strong D1 correlations, including CRP/SAA, IL-2/IL-10, IL-1ꞵ/TNF-α, IL-2/TNF-α, and IL-6/TNF-α, vs. CRP/SAA alone (Fig. 2).

### Inflammatory biomarkers are associated with TBI severity

The abilities of D1 and W2 inflammatory biomarkers to differentiate GCS 3–12 TBI, GCS 13–15 TBI, CT + TBI, CT- TBI, OC, and HC groups were examined. Thirteen D1 biomarkers differed significantly between GCS 3–12 vs. OC, GCS 3–12 vs. GCS 13–15, and CT + vs. CT- groups, which included CRP, SAA, IL-1ꞵ, IL-2, IL-4, IL-6, IL-10, IL-12p70, IL-15, IL-16, IL-17A, MCP-4, and TNF-α. Fifteen W2 biomarkers differed significantly across groups. Compared with D1 biomarker profiles, significant W2 biomarkers additionally included IL-5, IL-7, IP-10, MIP-1α, and MIP-1ꞵ, and did not include IL-12p70, IL-16, or MCP-4 (Table 2). Taken together, 10 inflammatory biomarkers (CRP, SAA, IL-1ꞵ, IL-2, IL-4, IL-6, IL-10, IL-15, IL-17A, TNF-α) demonstrated consistent diagnostic performance across clinico-radiographic TBI classification criteria at both D1 and W2 and were prioritized for assessments of prognostic ability.

**Table 2:** Inflammatory biomarker levels at day 1 and 2-weeks post-injury across TBI and control groups

Biomarker	Time Point	GCS 3–12 TBI vs. OC (fold change)	GCS 3–12 TBI vs. GCS 13–15 TBI (fold change)	GCS 13–15 TBI vs. OC (fold change)	GCS 13–15 TBI vs. HC (fold change)	CT + TBI vs. CT- TBI (fold change)
CRP	Day 1	**25.57/22.31 (9.6)**	**25.57/22.21 (10.3)**	22.21/22.31 (0.9)	**22.21/19.92 (4.9)**	**25.13/22.04 (8.5)**
	2-Week	**25.41/21.45 (15.6)**	**25.41/21.19 (18.6)**	21.19/21.45 (0.8)	**21.19/19.92 (2.4)**	**24.63/21.08 (11.7)**
SAA	Day 1	**27.73/23.01 (26.4)**	**27.73/23.51 (18.6)**	23.51/23.01 (1.4)	**23.51/20.96 (5.9)**	**27.50/23.20 (19.7)**
	2-Week	**26.46/21.83 (24.8)**	**26.46/21.82 (24.9)**	21.82/21.83 (1.0)	**21.82/20.96 (1.8)**	**24.78/21.77 (8.1)**
Eotaxin	Day 1	**7.05/7.36 (0.8)**	7.05/7.25 (0.9)	7.05/7.25 (0.9)	**7.25/7.73 (0.7)**	7.13/7.23 (0.9)
	2-Week	7.75/7.59 (1.1)	7.75/7.59 (1.1)	7.75/7.59 (1.1)	7.59/7.73 (0.9)	**7.80/7.53 (1.2)**
Eotaxin-3	Day 1	4.34/4.54 (0.9)	4.34/4.54 (0.9)	4.54/4.54 (1.0)	4.54/4.78 (0.8)	4.43/4.45 (1.0)
	2-Week	4.93/4.69 (1.2)	**4.93/4.60 (1.3)**	4.60/4.69 (0.9)	4.60/4.78 (0.9)	**4.91/4.49 (1.3)**
IFN-γ	Day 1	−1.99/−1.88 (0.9)	−1.99/−1.91 (0.9)	−1.91/−1.88 (1.0)	**−1.91/−1.26 (0.6)**	−2.01/−1.86 (0.9)
	2-Week	−0.77/−1.36 (1.5)	−0.77/−1.12 (1.3)	−1.12/−1.36 (1.2)	−1.12/−1.26 (1.1)	−0.97/−1.10 (1.1)
IL-1ɑ	Day 1	4.31/4.15 (1.1)	4.31/4.30 (1.0)	4.30/4.15 (1.1)	4.30/4.56 (0.8)	4.28/4.38 (0.9)
	2-Week	4.38/4.45 (1)	4.38/4.20 (1.1)	4.20/4.56 (0.8)	4.20/4.56 (0.8)	4.32/4.21 (1.1)
IL-1ꞵ	Day 1	**−0.30/−3.31 (8.1)**	**−0.30/−2.52 (4.7)**	−2.52/−3.31 (1.7)	**−2.52/−3.31 (1.7)**	**−0.72/−2.52 (3.5)**
	2-Week	**−2.06/−3.31 (2.4)**	**−2.06/−3.31 (2.4)**	−3.31/−3.31 (1.0)	−3.31/−3.31 (1.0)*	**−2.38/−3.31 (1.9)**
IL-2	Day 1	**−0.75/−3.10 (5.1)**	**−0.75/−2.83 (4.2)**	−2.83/−3.10 (1.2)	**−2.83/−3.82 (2.0)**	**−1.21/−2.95 (3.3)**
	2-Week	**−2.36/−3.59 (2.3)**	**−2.36/−3.64 (2.4)**	−3.64/−3.59 (1.0)	−3.65/−3.82 (1.1)	**−2.68/−3.63 (1.9)**
IL-4	Day 1	**−3.42/−5.68 (4.8)**	**−3.42/−5.44 (4.1)**	−5.44/−5.68 (1.2)	**−5.44/−6.06 (1.5)**	**−3.70/−5.44 (3.3)**
	2-Week	**−4.68/−5.88 (2.3)**	**−4.68/−6.06 (2.6)**	−6.06/−5.88 (0.9)	−6.06/−6.06 (1.0)	**−5.06/−6.06 (2.0)**
IL-5	Day 1	−1.15/−1.25 (1.1)	−1.15/−1.47 (1.2)	−1.47/−1.25 (0.9)	−1.47/−1.47 (1.0)	**-**1.47/−1.36 (0.9)
	2-Week	**−0.58/−1.07 (1.4)**	**−0.58/−1.18 (1.5)**	−1.18/−1.07 (0.9)	−1.18/−1.47 (1.2)	**−0.70/−1.18 (1.4)**
IL-6	Day 1	**6.29/4.33 (3.9)**	**6.29/4.39 (3.7)**	4.39/4.33 (1.0)	**4.39/0.57 (14.1)**	**6.29/4.07 (4.7)**
	2-Week	**4.47/1.92 (5.9)**	**4.47/1.41 (8.3)**	1.41/1.92 (0.7)	**1.41/0.57 (1.8)**	**3.84/1.29 (5.9)**
IL-7	Day 1	2.41/2.32 (1.1)	2.41/2.49 (0.9)	2.49/2.32 (1.1)	2.49/2.35 (1.1)	2.44/2.42 (1.0)
	2-Week	**3.22/2.57 (1.6)**	**3.22/2.54 (1.6)**	2.54/2.57 (1.0)	2.54/2.35 (1.1)	**3.13/2.25 (1.8)**
IL-10	Day 1	**2.78/−0.12 (7.5)**	**2.78/−0.24 (8.1)**	−0.24/−0.12 (0.9)	**−0.24/−0.69 (1.4)**	**2.36/−0.31 (6.4)**
	2-Week	**0.68/−0.81 (2.8)**	**0.68/−0.67 (2.5)**	−0.67/−0.81 (1.1)	−0.67/−0.69 (1.0)	**0.32/−0.71 (2.0)**
IL-12/23p40	Day 1	**6.52/6.85 (0.8)**	6.52/6.68 (0.9)	6.68/6.85 (0.9)	**6.68/7.49 (0.6)**	6.53/6.70 (0.9)
	2-Week	7.08/7.23 (0.9)	7.08/7.14 (1.0)	7.14/7.23 (0.9)	**7.14/7.49 (0.8)**	7.15/7.09 (1.0)
IL-12p70	Day 1	**−0.72/−1.74 (2.0)**	**−0.72/−1.57 (1.8)**	−1.57/−1.74 (1.1)	−1.57/−1.69 (1.1)	**−0.96/−1.54 (1.5)**
	2-Week	**−1.21/−1.93 (1.6)**	−1.21/−1.62 (1.3)	−1.62/−1.93 (1.2)	−1.62/−1.69 (1.0)	−1.37/−1.56 (1.1)
IL-15	Day 1	**2.47/1.67 (1.7)**	**2.47/1.86 (1.5)**	1.86/1.67 (1.1)	**1.86/1.57 (1.2)**	**2.29/1.84 (1.4)**
	2-Week	**1.88/1.44 (1.4)**	**1.88/1.50 (1.3)**	1.50/1.44 (1.0)	1.50/1.57 (1.0)	**1.69/1.49 (1.1)**
IL-16	Day 1	**7.90/7.14 (1.7)**	**7.90/7.31 (1.5)**	7.31/7.14 (1.1)	7.31/7.33 (1.0)	**7.73/7.32 (1.3)**
	2-Week	7.29/7.09 (1.1)	7.29/7.15 (1.1)	7.15/7.09 (1.0)	7.14/7.33 (0.9)	7.22/7.14 (1.1)
IL-17A	Day 1	**−0.24/−1.63 (2.6)**	**−0.24/−1.37 (2.2)**	−1.37/−1.63 (1.2)	−1.37/−1.34 (1.0)	**−0.52/−1.36 (1.8)**
	2-Week	**0.22/−1.64 (3.6)**	**0.22/−1.36 (3.0)**	−1.36/−1.64 (1.2)	−1.36/−1.34 (1.0)	**−0.29/−1.32 (2.0)**
IP-10	Day 1	7.82/7.95 (0.9)	7.82/7.68 (1.1)	7.68/7.95 (0.8)	**7.68/8.11 (0.7)**	7.80/7.68 (1.1)
	2-Week	**8.57/8.20 (1.3)**	**8.57/8.18 (1.3)**	8.18/8.20 (1.0)	8.18/8.11 (1.0)	**8.52/8.11 (1.3)**
MCP-1	Day 1	6.99/6.71 (1.2)	**6.99/6.56 (1.3)**	6.56/6.71 (0.9)	6.56/6.64 (0.9)	**6.89/6.52 (1.3)**
	2-Week	6.90/6.72 (1.1)	**6.90/6.66 (1.2)**	6.66/6.72 (1.0)	6.66/6.64 (1.0)	**6.87/6.62 (1.2)**
MCP-4	Day 1	**5.44/6.16 (0.6)**	**5.44/6.23 (0.6)**	6.23/6.16 (1.0)	6.23/6.14 (1.1)	**5.68/6.10 (0.7)**
	2-Week	**6.78/6.45 (1.3)**	6.78/6.49 (1.2)	6.49/6.45 (1.0)	6.49/6.14 (1.3)	**6.76/6.36 (1.3)**
MDC	Day 1	9.45/9.57 (0.9)	9.45/9.63 (0.9)	9.63/9.57 (1.0)	**9.63/9.93 (0.8)**	**9.44/9.73 (0.8)**
	2-Week	**9.68/9.90 (0.9)**	9.68/9.85 (0.9)	9.85/9.90 (1.0)	9.85/9.93 (1.0)	9.71/9.90 (0.9)
MIP-1α	Day 1	4.11/3.96 (1.1)	4.11/3.98 (1.1)	3.98/3.96 (1.0)	**3.98/4.15 (0.9)**	4.05/3.98 (1.0)
	2-Week	**4.39/4.06 (1.3)**	**4.39/4.00 (1.3)**	4.0/4.06 (1.0)	4.00/4.15 (0.9)	**4.26/3.99 (1.2)**
MIP-1ꞵ	Day 1	**6.64/6.48 (1.1)**	6.64/6.38 (1.2)	6.38/6.48 (1.1)	6.38/6.28 (1.1)	6.55/6.45 (1.1)
	2-Week	**6.73/6.42 (1.2)**	**6.73/6.23 (1.4)**	6.23/6.42 (1.1)	6.23/6.28 (1.0)	**6.62/6.17 (1.4)**
TARC	Day 1	6.61/6.57 (1.0)	6.61/6.86 (0.8)	6.86/6.57 (1.2)	6.86/6.43 (1.3)	6.63/6.91 (0.8)
	2-Week	6.79/7.02 (0.9)	6.79/6.68 (1.1)	6.68/7.02 (0.8)	6.68./6.43 (1.2)	6.84/6.53 (1.2)
TNF-ɑ	Day 1	**0.98/−0.64 (3.1)**	**0.98/−0.73 (3.3)**	−0.73/−0.64 (0.9)	−0.73/−0.60 (0.9)	**0.45/−0.82 (2.4)**
	2-Week	**−0.15/−0.83 (1.6)**	**−0.15/−0.85 (1.6)**	−0.85/−0.83 (1.0)	−0.85/−0.60 (0.8)	**−0.32/−0.93 (1.5)**
TNF-β	Day 1	−1.60/−1.60 (1.0)	−1.60/−1.60 (1.0)	−1.60/−1.60 (1.0)	−1.60/−1.60 (1.0)	**−1.6/−1.12 (0.7)**
	2-Week	−1.60/−1.60 (1.0)	−1.60/−1.60 (1.0)	−1.60/−1.60 (1.0)	−1.60/−1.60 (1.0)	−1.16/−1.12 (0.7)

Caption: Plasma inflammatory biomarkers levels at D1 and W2 post-injury in TBI (GCS 3–12, GCS 13–15, CT +, CT-) and control groups were compared. Median log_2_ values are shown for each group. Fold changes were calculated using median log_2_ values and converted back to raw scale (shown in parentheses). The Bonferroni correction was performed and results statistically significant at *p* < 0.0018 are shown in bold. ^*^Statistically significant due to differences in distribution of data despite equal means. CRP = C-reactive protein; CT = computed tomography; HC = healthy control; GCS = Glasgow Coma Scale; IFN = interferon; IL = interleukin; IP = interferon-gamma induced protein; MCP = monocyte chemoattractant protein; MDC = macrophage-derived chemokine; MIP = macrophage inflammatory protein; OC = orthopedic trauma control; SAA = serum amyloid A; TARC = thymus and activation regulated chemokine; TBI = traumatic brain injury; TNF = tumor necrosis factor

Inflammatory biomarkers did not differ significantly between GCS 13–15 vs. OC at D1 or W2. SAA, CRP, IL-1ꞵ, IL-2, IL-4, IL-10, and IL-15 differed significantly between GCS 13–15 vs. HC on D1. CRP, SAA, IL-1ꞵ, and IL-6 differed significantly between GCS 13–15 vs. HC at W2.

**Fig. 3:**
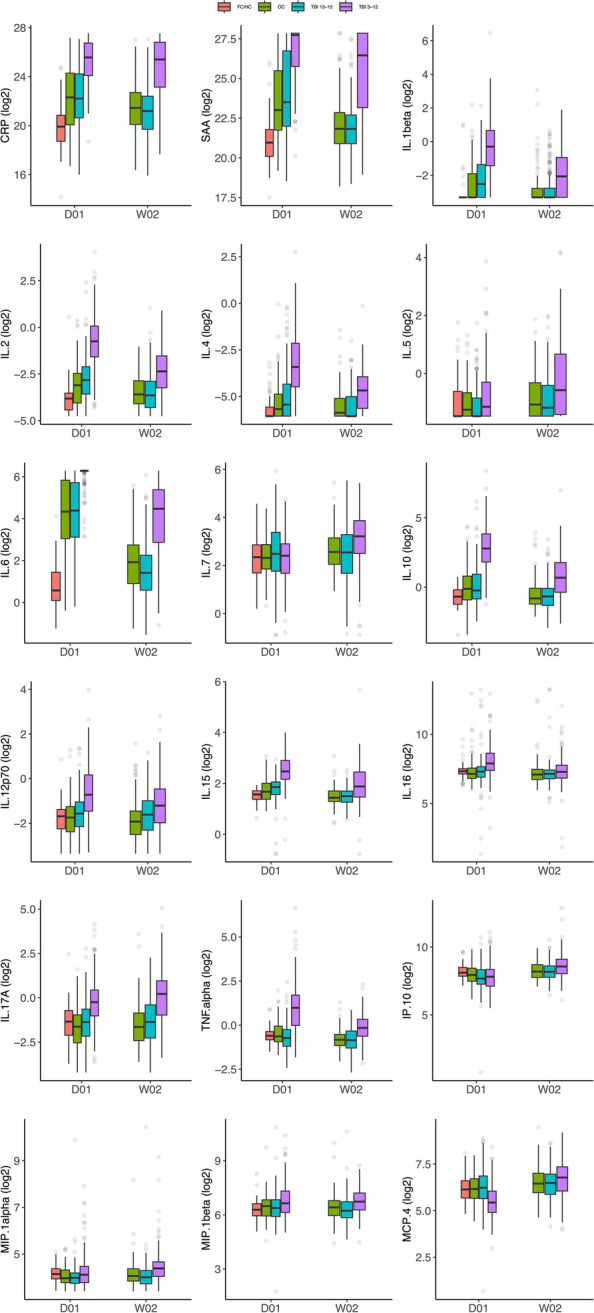
Inflammatory Biomarker Levels at Day 1 and 2 Weeks Post-Injury Across TBI and Control Groups. Boxplots constructed from log_2_-transformed biomarker levels (median, quartile 1 (Q1) to quartile 3 (Q3)) in healthy controls (red), orthopedic trauma controls (green), GCS 13–15 TBI (blue), and GCS 3–12 TBI (violet). Biomarkers able to differentiate group comparisons across GCS 3–12 TBI vs. OC, GCS 3–12 vs. GCS 13–15 TBI, and CT + vs. CT- TBI at day 1 or 2-weeks are shown. Vertical lines indicate minima and maxima without outliers. Dots represent outliers, defined as lower than 1.5 times the interquartile range below Q1 or higher than 1.5 times the interquartile range above Q3. CT = computed tomography; D01 = day 1; FC/HC = friend control/healthy control; GCS = Glasgow Coma Scale; IL = interleukin; IP = interferon-gamma induced protein; MCP = monocyte chemoattractant protein; MIP = macrophage inflammatory protein; OC = orthopedic trauma control; SAA = serum amyloid A; TBI = traumatic brain injury; TNF = tumor necrosis factor; W02 = 2-week

Inflammatory biomarkers associated with TBI were generally higher levels in subjects with more severe injuries, with the exception of MCP-4 which showed an inverse relationship with TBI severity (Table 2, Fig. 3).

Of these biomarkers, the majority showed moderate (AUC 0.7–0.8) to strong (AUC 0.8–0.9) discriminatory ability at D1 and W2 across three comparisons of interest: GCS 3–12 vs. OC, GCS 3–12 vs. 13–15, and CT + vs. CT-. CRP, SAA, IL-1β, IL-2, IL-4, IL-6, IL-10, IL-15, IL-17A, and TNF-α showed strong discrimination between GCS 3–12 TBI vs. OC (D1/W2 AUC: 0.82/0.85, 0.83/0.83, 0.89/0.73, 0.89/0.77, 0.86/0.70, 0.89/0.82, 0.89/0.78, 0.88/0.72, 0.81/0.79, and 0.85/0.76, respectively). These biomarkers demonstrated similar performance for distinguishing GCS 3–12 TBI from GCS 13–15 TBI (D1/W2 AUC: 0.82/0.88, 0.80/0.85, 0.84/0.72, 0.87/0.77, 0.81/0.73, 0.87/0.87, 0.89/0.78, 0.86/0.72, 0.75/0.75, and 0.87/0.75, respectively), with the exception of IL-17A. CRP, IL-2, IL-6, IL-10, and TNF-α showed moderate to strong discrimination of radiographic TBI severity (D1/W2 AUC: 0.78/0.83, 0.82/0.72, 0.81/0.82, 0.81/0.72, and 0.81/0.71, respectively). IL-6, CRP, and SAA showed the strongest overall discriminatory ability across both timepoints (Table 3).

**Table 3:** Discriminatory performance of inflammatory biomarkers for TBI diagnosis and severity

Biomarker	Time Point	GCS 3–12 TBI vs. OC AUC (95% CI)	GCS 3–12 TBI vs. 13–15 TBI AUC (95% CI)	GCS 13–15 TBI vs. OC AUC (95% CI)	GCS 13–15 TBI vs. HC AUC (95% CI)	CT + TBI vs. CT- TBI AUC (95% CI)
CRP	Day 1	**0.82 (0.77–0.87)**	**0.82 (0.78–0.86)**	0.48 (0.41–0.56)	**0.77 (0.71–0.83)**	**0.78 (0.73–0.83)**
	2-Week	**0.85 (0.80–0.90)**	**0.88 (0.84–0.91)**	0.55 (0.48–0.62)	0.66 (0.59–0.73)	**0.83 (0.79–0.87)**
SAA	Day 1	**0.83 (0.78–0.89)**	**0.80 (0.75–0.84)**	0.54 (0.47–0.62)	**0.83 (0.78–0.88)**	**0.77 (0.72–0.82)**
	2-Week	**0.83 (0.78–0.88)**	**0.85 (0.81–0.89)**	0.52 (0.44–0.59)	0.67 (0.59–0.74)	**0.79 (0.74–0.83)**
Eotaxin	Day 1	0.63 (0.56–0.69)	0.59 (0.53–0.65)	0.54 (0.48–0.59)	**0.70 (0.63–0.77)**	0.53 (0.47–0.59)
	2-Week	0.53 (0.46–0.60)	0.55 (0.49–0.61)	0.52 (0.45–0.59)	0.56 (0.48–0.64)	0.64 (0.59–0.70)
Eotaxin-3	Day 1	0.58 (0.51–0.65)	0.55 (0.45–0.57)	0.47 (0.40–0.54)	0.60 (0.53–0.68)	0.48 (0.42–0.54)
	2-Week	0.59 (0.52–0.66)	0.65 (0.59–0.70)	0.56 (0.49–0.63)	0.57 (0.50–0.65)	0.68 (0.63–0.74)
IFN-γ	Day 1	0.54 (0.47–0.61)	0.53 (0.47–0.59)	0.51 (0.44–0.57)	**0.70 (0.63–0.76)**	0.56 (0.51–0.62)
	2-Week	0.56 (0.49–0.63)	0.52 (0.46–0.58)	0.56 (0.48–0.63)	0.56 (0.48–0.63)	0.50 (0.44–0.56)
IL-1ɑ	Day 1	0.52 (0.44–0.59)	0.52 (0.46–0.58)	0.49 (0.42–0.56)	0.58 (0.50–0.66)	0.51 (0.45–0.57)
	2-Week	0.52 (0.44–0.59)	0.53 (0.47–0.59)	0.54 (0.47–0.61)	0.58 (0.50–0.66)	0.50 (0.44–0.56)
IL-1ꞵ	Day 1	**0.89 (0.85–0.93)**	**0.84 (0.80–0.88)**	0.6 (0.54–0.67)	**0.78 (0.73–0.83)**	**0.76 (0.71–0.81)**
	2-Week	**0.73 (0.67–0.79)**	**0.72 (0.67–0.77)**	0.51 (0.45–0.57)	0.62 (0.57–0.67)	**0.70 (0.64–0.75)**
IL-2	Day 1	**0.89 (0.84–0.93)**	**0.87 (0.83–0.90)**	0.57 (0.50–0.64)	**0.78 (0.72–0.84)**	**0.82 (0.78–0.86)**
	2-Week	**0.77 (0.71–0.82)**	**0.77 (0.72–0.82)**	0.52 (0.45–0.59)	0.60 (0.53–0.68)	**0.72 (0.66–0.77)**
IL-4	Day 1	**0.86 (0.81–0.90)**	**0.81 (0.77–0.85)**	0.55 (0.49–0.62)	0.67 (0.60–0.73)	**0.74 (0.69–0.79)**
	2-Week	**0.70 (0.64–0.77)**	**0.73 (0.68–0.78)**	0.54 (0.48–0.61)	0.55 (0.49–0.62)	0.69 (0.64–0.75)
IL-5	Day 1	0.53 (0.46–0.59)	0.59 (0.53–0.64)	0.57 (0.51–0.64)	0.50 (0.43–0.58)	0.49 (0.43–0.55)
	2-Week	0.63 (0.56–0.69)	0.65 (0.59–0.71)	0.53 (0.46–0.60)	0.57 (0.50–0.65)	0.61 (0.55–0.66)
IL-6	Day 1	**0.89 (0.84–0.93)**	**0.87 (0.84–0.91)**	0.52 (0.45–0.59)	**0.95 (0.93–0.98)**	**0.81 (0.76–0.86)**
	2-Week	**0.82 (0.77–0.88)**	**0.87 (0.83–0.91)**	0.58 (0.50–0.65)	0.66 (0.59–0.74)	**0.82 (0.76–0.86)**
IL-7	Day 1	0.50 (0.43–0.57)	0.55 (0.49–0.61)	0.55 (0.48–0.61)	0.57 (0.50–0.64)	0.48 (0.42–0.55)
	2-Week	0.66 (0.59–0.72)	0.65 (0.60–0.71)	0.53 (0.47–0.60)	0.54 (0.47–0.61)	0.69 (0.63–0.74)
IL-10	Day 1	**0.89 (0.83–0.93)**	**0.89 (0.86–0.93)**	0.50 (0.43–0.57)	0.66 (0.59–0.73)	**0.81 (0.76–0.85)**
	2-Week	**0.78 (0.72–0.84)**	**0.78 (0.73–0.83)**	0.52 (0.45–0.59)	0.50 (0.42–0.57)	**0.72 (0.67–0.77)**
IL-12/23p40	Day 1	0.63 (0.57–0.70)	0.56 (0.50–0.62)	0.57 (0.51–0.64)	**0.79 (0.73–0.84)**	0.56 (0.50–0.62)
	2-Week	0.55 (0.48–0.62)	0.53 (0.47–0.59)	0.53 (0.46–0.60)	0.64 (0.56–0.71)	0.52 (0.46–0.58)
IL-12p70	Day 1	**0.77 (0.71–0.82)**	**0.72 (0.66–0.77)**	0.57 (0.50–0.64)	0.57 (0.50–0.65)	0.66 (0.60–0.72)
	2-Week	0.67 (0.60–0.74)	0.61 (0.55–0.66)	0.57 (0.50–0.64)	0.56 (0.48–0.63)	0.58 (0.52–0.63)
IL-15	Day 1	**0.88 (0.83–0.92)**	**0.86 (0.83–0.90)**	0.58 (0.50–0.65)	**0.71 (0.65–0.77)**	**0.77 (0.72–0.82)**
	2-Week	**0.72 (0.66–0.78)**	**0.72 (0.67–0.77)**	0.50 (0.43–0.57)	0.55 (0.47–0.63)	0.69 (0.64–0.75)
IL-16	Day 1	**0.74 (0.68–0.80)**	**0.72 (0.66–0.77)**	0.57 (0.49–0.64)	0.50 (0.43–0.58)	0.65 (0.59–0.71)
	2-Week	0.58 (0.52–0.65)	0.58 (0.52–0.64)	0.51 (0.44–0.58)	0.62 (0.55–0.70)	0.59 (0.53–0.65)
IL-17A	Day 1	**0.81 (0.75–0.86)**	**0.75 (0.70–0.80)**	0.58 (0.51–0.65)	0.49 (0.42–0.57)	0.69 (0.63–0.74)
	2-Week	**0.79 (0.73–0.85)**	**0.75 (0.70–0.80)**	0.56 (0.49–0.62)	0.48 (0.40–0.55)	0.67 (0.62–0.73)
IP-10	Day 1	0.56 (0.49–0.63)	0.51 (0.45–0.57)	0.58 (0.51–0.65)	0.68 (0.61–0.74)	0.52 (0.46–0.58)
	2-Week	0.65 (0.59–0.72)	0.67 (0.61–0.72)	0.51 (0.44–0.59)	0.51 (0.43–0.59)	0.67 (0.61–0.72)
MCP-1	Day 1	0.61 (0.54–0.67)	0.65 (0.59–0.71)	0.57 (0.50–0.64)	0.54 (0.46–0.61)	0.61 (0.56–0.67)
	2-Week	0.57 (0.50–0.64)	0.61 (0.56–0.67)	0.56 (0.49–0.63)	0.51 (0.43–0.59)	0.63 (0.57–0.69)
MCP-4	Day 1	**0.73 (0.66–0.79)**	**0.73 (0.67–0.78)**	0.53 (0.46–0.60)	0.53 (0.45–0.61)	0.63 (0.57–0.69)
	2-Week	0.57 (0.5–0.64)	0.58 (0.52–0.64)	0.49 (0.42–0.56)	0.60 (0.52–0.67)	0.63 (0.57–0.69)
MDC	Day 1	0.57 (0.49–0.64)	0.56 (0.50–0.62)	0.50 (0.43–0.57)	**0.70 (0.64–0.77)**	0.63 (0.57–0.69)
	2-Week	0.64 (0.57–0.70)	0.58 (0.52–0.64)	0.56 (0.49–0.62)	0.58 (0.51–0.66)	0.58 (0.52–0.64)
MIP-1α	Day 1	0.56 (0.49–0.63)	0.59 (0.53–0.65)	0.47 (0.40–0.54)	0.65 (0.57–0.72)	0.55 (0.50–0.61)
	2-Week	0.65 (0.59–0.72)	0.71 (0.66–0.77)	0.57 (0.50–0.64)	0.62 (0.55–0.69)	0.68 (0.62–0.73)
MIP-1ꞵ	Day 1	0.60 (0.53–0.67)	0.62 (0.56–0.68)	0.53 (0.46–0.60)	0.55 (0.47–0.63)	0.57 (0.51–0.63)
	2-Week	0.63 (0.56–0.70)	0.68 (0.62–0.73)	0.56 (0.49–0.63)	0.50 (0.43–0.58)	0.65 (0.60–0.71)
TARC	Day 1	0.49 (0.42–0.56)	0.55 (0.49–0.61)	0.53 (0.46–0.60)	0.61 (0.54–0.68)	0.56 (0.50–0.62)
	2-Week	0.56 (0.49–0.63)	0.50 (0.44–0.56)	0.56 (0.49–0.63)	0.60 (0.53–0.68)	0.54 (0.48–0.60)
TNF-ɑ	Day 1	**0.85 (0.80–0.89)**	**0.87 (0.83–0.90)**	0.57 (0.50–0.64)	0.56 (0.49–0.63)	**0.81 (0.77–0.86)**
	2-Week	**0.76 (0.70–0.82)**	**0.75 (0.70–0.80)**	0.50 (0.43–0.57)	0.63 (0.56–0.69)	**0.71 (0.66–0.76)**
TNF-β	Day 1	0.51 (0.45–0.54)	0.47 (0.42–0.53)	0.54 (0.48–0.59)	0.68 (0.60–0.75)	0.49 (0.44–0.54)
	2-Week	0.45 (0.39–0.52)	0.45 (0.40–0.50)	0.50 (0.44–0.57)	0.62 (0.54–0.70)	0.45 (0.39–0.50)

Caption: The ability of each inflammatory biomarker to differentiate between TBI and control groups at D1 and W2 is shown using area under the receiver-operating characteristic curve (AUC) and associated 95% confidence interval (CI). Bolded values signify AUC ≥ 0.7. CRP = C-reactive protein; CT = computed tomography; D1 = day 1, 0–24 h post-injury; GCS = Glasgow Coma Scale; HC = healthy control; IFN = interferon; IL = interleukin; IP = interferon-gamma induced protein; MCP = monocyte chemoattractant protein; MIP = macrophage inflammatory protein; OC = orthopedic trauma control; SAA = serum amyloid A; TARC = thymus and activation regulated chemokine; TBI = traumatic brain injury; TNF = tumor necrosis factor; W2 = 2-week

Biomarkers that did not reach moderate discriminatory ability were IL-7 (D1, W2), MIP-1ꞵ (D1, W2), IL-12p70 (W2), and IL-16 (W2). Biomarkers did not discriminate GCS 13–15 TBI vs. OC at D1 and W2. CRP, SAA, IL-1ꞵ, IL-6, and IL-15 discriminated GCS 13–15 TBI vs. HC on D1 (Table 3).

### Subacute inflammatory biomarkers predict 6-month unfavorable outcomes

The prognostic associations of inflammatory biomarkers with 6-month functional outcomes in TBI subjects was examined. Of 369 TBI subjects, 70 (19.0%) had unfavorable functional outcomes (GOSE 1–4) and 299 (81.0%) had favorable outcomes (GOSE 5–8). Clinical indicators of more severe initial injury, including elevated plasma D1 GFAP levels, ICU admission, major extracranial injury, traumatic intracranial injuries on CT, and higher Marshall CT scores, showed higher incidences in subjects with 6-month unfavorable outcomes (Supplementary Table S4).

On multivariable logistic regression models, D1 inflammatory biomarkers were not significantly associated with 6-month unfavorable outcomes after multiple comparisons correction (Table 4).

**Table 4:** Logistic regression models for acute (Day 1) and subacute (2-Week) inflammatory biomarkers as predictors of 6-month unfavorable outcome (GOSE 1–4 vs. 5–8)

	**Day 1 Biomarker as Predictor**				**2-Week Biomarker as Predictor**			
**Biomarker**	**Raw OR (95% CI)**	**AOR (95% CI)**	**Uncorrected *p*-value**	**Corrected *p*-value**	**Raw OR (95% CI)**	**AOR (95% CI)**	**Uncorrected *p*-value**	**Corrected *p*-value**
CRP	1.39 (1.22–1.59)	1.02 (0.83–1.26)	0.820	0.915	2.18 (1.80–2.63)	**1.77 (1.38–2.28)**	< 0.001	** < 0.001**
SAA	1.36 (1.19–1.57)	1.00 (0.78–1.30)	0.970	0.970	2.29 (1.89–2.77)	**2.24 (1.65–3.05)**	< 0.001	** < 0.001**
Eotaxin	0.77 (0.53–1.10)	0.93 (0.54–1.60)	0.787	0.915	1.36 (0.93–2.00)	0.93 (0.52–1.65)	0.806	0.870
Eotaxin-3	0.62 (0.44–0.88)	0.56 (0.34–0.93)	0.025	0.112	1.17 (0.92–1.49)	0.73 (0.44–1.20)	0.213	0.338
IFN-γ	0.94 (0.77–1.15)	1.04 (0.79–1.37)	0.781	0.915	0.94 (0.76–1.16)	0.87 (0.67–1.13)	0.287	0.369
IL-1α	0.86 (0.68–1.10)	0.76 (0.52–1.10)	0.146	0.429	0.83 (0.63–1.08)	0.8 (0.55–1.15)	0.225	0.338
IL-1β	1.69 (1.42–2.00)	1.12 (0.86–1.46)	0.397	0.670	1.74 (1.42–2.14)	1.28 (0.94–1.76)	0.120	0.216
IL-2	2.01 (1.66–2.44)	1.46 (1.09–1.96)	0.011	0.112	2.10 (1.66–2.65)	1.45 (1.04–2.00)	0.027	0.061
IL-4	1.54 (1.32–1.79)	1.14 (0.88–1.46)	0.318	0.572	1.56 (1.23–1.97)	1.14 (0.81–1.61)	0.440	0.531
IL-5	1.35 (1.01–1.81)	0.89 (0.60–1.32)	0.567	0.806	1.99 (1.57–2.51)	**1.53 (1.12–2.10)**	0.007	**0.024**
IL-6	4.13 (2.25–7.59)	1.54 (0.78–3.06)	0.212	0.440	2.76 (2.18–3.50)	**2.21 (1.59–3.08)**	< 0.001	** < 0.001**
IL-7	0.73 (0.57–0.93)	0.61 (0.41–0.90)	0.014	0.112	1.33 (1.07–1.66)	0.86 (0.62–1.21)	0.399	0.490
IL-10	1.87 (1.58–2.21)	1.50 (1.17–1.94)	0.002	0.054	1.96 (1.59–2.40)	**1.41 (1.07–1.84)**	0.013	**0.032**
IL-12/23p40	0.79 (0.62–1.00)	0.80 (0.57–1.11)	0.175	0.430	0.68 (0.51–0.90)	0.70 (0.48–1.03)	0.071	0.137
IL-12p70	1.76 (1.40–2.21)	1.31 (0.94–1.83)	0.107	0.361	1.68 (1.31–2.13)	1.49 (1.04–2.14)	0.030	0.062
IL-15	5.65 (3.40–9.41)	1.29 (0.66–2.51)	0.455	0.682	7.50 (4.45–12.65)	**3.43 (1.71–6.87)**	0.001	**0.005**
IL-16	1.29 (1.05–1.60)	0.96 (0.69–1.33)	0.813	0.915	1.36 (1.05–1.75)	1.31 (0.92–1.86)	0.129	0.218
IL-17A	1.82 (1.47–2.24)	1.40 (1.05–1.87)	0.024	0.112	2.31 (1.83–2.90)	**1.97 (1.46–2.64)**	< 0.001	** < 0.001**
IP-10	1.08 (0.83–1.41)	0.99 (0.67–1.46)	0.958	0.970	1.78 (1.27–2.48)	0.98 (0.62–1.57)	0.948	0.948
MCP-1	1.56 (1.24–1.96)	1.27 (0.91–1.76)	0.159	0.429	1.90 (1.29–2.80)	1.33 (0.79–2.25)	0.281	0.369
MCP-4	0.52 (0.39–0.70)	0.56 (0.35–0.90)	0.017	0.112	1.37 (1.03–1.83)	0.88 (0.59–1.32)	0.531	0.597
MDC	0.89 (0.58–1.36)	1.09 (0.50–2.37)	0.821	0.915	0.26 (0.15–0.44)	**0.39 (0.19–0.80)**	0.010	**0.027**
MIP-1α	1.60 (1.12–2.27)	1.51 (0.92–2.47)	0.100	0.361	2.57 (1.65–4.01)	**1.77 (1.17–2.68)**	0.007	**0.024**
MIP-1β	1.35 (1.01–1.80)	1.04 (0.60–1.58)	0.847	0.915	1.94 (1.36–2.76)	1.36 (0.81–2.30)	0.249	0.354
TARC	0.82 (0.66–1.04)	0.79 (0.55–1.14)	0.204	0.440	0.76 (0.61–0.95)	**0.67 (0.49–0.91)**	0.010	**0.027**
TNF-α	1.96 (1.59–2.40)	1.19 (0.87–1.61)	0.248	0.548	4.14 (2.70–6.34)	**2.35 (1.35–4.06)**	0.002	**0.009**
TNF-β	1.23 (0.63–2.40)	1.36 (0.61–3.05)	0.448	0.682	0.54 (0.23–1.24)	0.91 (0.32–2.57)	0.854	0.887

Caption: Univariate and multivariable logistic regression models for unfavorable 6-month functional outcomes (GOSE 1–4 vs. 5–8) are shown for plasma biomarkers measured on day 1 and at 2-weeks. Unadjusted odds ratios (OR), adjusted odds ratios (AOR), and 95% confidence intervals (CI) represent the change in odds per log_2_-unit increase in biomarker level. Multivariable models were fit separately for each biomarker and adjusted for age (per-year), sex (male/female), major extracranial injury (AIS ≥ 3 for extracranial body systems; yes/no), emergency department arrival GCS (3–12 vs. 13–15), psychiatric history (yes/no), Marshall CT score (1 vs. 2, 3–4, 5–6), and log_2_-transformed day 1 GFAP levels. Uncorrected *p*-values are shown for multivariable models, and were corrected for multiple comparisons using the Benjamini–Hochberg method. Statistically significant results after multiple comparisons correction are shown in bold. AIS = Abbreviated Injury Scale; CRP = C-reactive protein; CT = computed tomography; GFAP = glial fibrillary acidic protein; GOSE = Glasgow Outcome Scale-Extended; IFN = interferon; IL = interleukin; IP = interferon-gamma induced protein; MCP = monocyte chemoattractant protein; MDC = macrophage-derived chemokine; MIP = macrophage inflammatory protein; SAA = serum amyloid A; TARC = thymus and activation regulated chemokine; TNF = tumor necrosis factor

At W2, 7 of the 10 biomarkers of interest identified in our diagnostic analyses (i.e. differentiated across clinico-radiographic TBI criteria at both D1 and W2) emerged as significant multivariable predictors of 6-month unfavorable outcomes after multiple comparisons correction: IL-15 (AOR: 3.43, 95% CI [1.71–6.87], *p* = 0.005), IL-6 (AOR: 2.21 [1.59–3.08], *p* < 0.001), IL-17A: (1.97 [1.46–2.64], *p* < 0.001), CRP (AOR: 1.77 [1.38–2.28], *p* < 0.001), SAA (AOR: 2.24 [1.65–3.05], *p* < 0.001), TNF-α (AOR: 2.35 [1.35–4.06], *p* = 0.009), and IL-10 (AOR: 1.41 [1.07–1.84], *p* = 0.032) (Table 4). Additional W2 biomarkers associated with 6-month unfavorable outcomes were IL-5, MDC, MIP-1α, and TARC (Table 4).

Exploratory analyses assessing potential sex-related differences in the association of 10 W2 biomarkers with 6-month unfavorable outcomes showed no significant biomarker-by-sex interactions, except for TNF-α, for which the association with unfavorable outcomes appeared stronger in females (nominal *p* = 0.014). (Supplementary Table S2).

Hospitalization status at the time of the W2 blood draw was significantly associated with 6-month unfavorable outcomes (*p* < 0.001; Supplementary Table S3). After adjusting for W2 hospitalization status and correcting for multiple comparisons, 5 W2 biomarkers remained as significant predictors of unfavorable outcomes: IL-15 (AOR: 2.26 95% CI [1.14–4.49], *p* = 0.040), SAA (AOR: 1.91 [1.37–2.67], *p* < 0.001), IL-6 (AOR: 1.80 [1.25–2.61], *p* = 0.007), IL-17A (AOR: 1.72 [1.24–2.39], *p* = 0.005), CRP (AOR: 1.40 [1.06–1.85], *p* = 0.040) (Table 5).

**Table 5:** Sensitivity models for subacute (2-Week) inflammatory biomarkers as predictors of 6-month unfavorable outcome (GOSE 1–4 vs. 5–8) adjusting for 2-week hospitalization status

	**2-Week Biomarker as Predictor**	
**Biomarker**	**AOR (95% CI)**	**Corrected *p*-value**
CRP	**1.40 (1.06–1.85)**	**0.040**
SAA	**1.91 (1.37–2.67)**	** < 0.001**
IL-1β	1.08 (0.75–1.54)	0.770
IL-2	1.13 (0.78–1.64)	0.660
IL-4	1.04 (0.71–1.54)	0.829
IL-6	**1.80 (1.25–2.61)**	**0.007**
IL-10	1.20 (0.88–1.63)	0.373
IL-15	**2.26 (1.14–4.49)**	**0.040**
IL-17A	**1.72 (1.24–2.39)**	**0.005**
TNF-α	1.71 (0.93–3.15)	0.140

Caption: Multivariable logistic regression results for 6-month unfavorable outcomes (GOSE 1–4 vs. 5–8) are shown for plasma biomarkers measured at 2-weeks. Adjusted odds ratios (AOR) and 95% confidence intervals (CI) represent the change in odds per log_2_-unit increase in biomarker level. Models were fit separately for each biomarker and adjusted for age (per-year), sex (male/female), major extracranial injury (AIS ≥ 3 for extracranial body systems; yes/no), emergency department arrival GCS (3–12 vs. 13–15), psychiatric history (yes/no), Marshall CT score (1 vs. 2, 3–4, 5–6), log_2_-transformed day 1 GFAP levels (per-unit), and hospitalization status at 2 weeks (not hospitalized, hospitalized not in ICU, hospitalized in ICU). *P*-values were corrected for multiple comparisons using the Benjamini–Hochberg method. Statistically significant results are shown in bold. AIS = Abbreviated Injury Scale; CRP = C-reactive protein; CT = computed tomography; GCS = Glasgow Coma Scale; GFAP = glial fibrillary acidic protein; GOSE = Glasgow Outcome Scale-Extended; ICU = intensive care unit; IL = interleukin; SAA = serum amyloid A; TNF = tumor necrosis factor

### Inflammatory biomarkers did not predict 6-month incomplete recovery on multivariable models

The prognostic associations between inflammatory biomarkers and 6-month incomplete vs. complete functional recovery (GOSE 1–7 vs. GOSE 8) were examined in TBI subjects. Demographic and clinical characteristics between TBI subjects with incomplete and complete functional recovery are shown in Supplementary Table S4. On multivariable models, inflammatory biomarkers at D1 and W2 did not show significant associations with 6-month incomplete functional recovery after multiple comparisons correction (Supplementary Tables S5).

## Discussion

Inflammatory cascades contribute to secondary injury after traumatic insults to the brain and body and are also involved in tissue repair and recovery. Determining and validating priority inflammatory biomarkers with diagnostic and prognostic value will aid in the assessment of TBI, enable detection of secondary injury, and improve prediction of long-term outcomes. In the present multicenter prospective cohort study, we analyze the diagnostic and prognostic capabilities of blood-based inflammatory markers across 369 TBI subjects, 100 OCs, and 69 HCs. We confirm the utility of previously identified profiles of plasma inflammatory biomarkers in differentiating clinical and radiographic severity after TBI at D1 and W2 post-injury. Importantly, after rigorous adjustment of socio-demographic and clinical injury variables and multiple comparisons correction, our study establishes that subacute (W2) levels of 7 inflammatory biomarkers are associated with 6-month unfavorable functional outcomes.

### Acute and subacute inflammatory biomarkers discriminate clinical and radiographic TBI severity

Our study showed that GCS 3–12 TBI subjects demonstrated significantly elevated plasma inflammatory biomarker levels when compared to OCs and HCs despite slight differences in time to sample collection in the GCS 3–12 TBI subjects. We do not believe these differences in time to sample collection within 24 h account for the observed increases in inflammatory biomarker expression, which is supported by similar patterns of elevation in GCS 3–12 TBI subjects at W2. Biomarkers with the strongest associations were IL-6, CRP, and SAA, which retained AUCs > 0.8 for differentiating clinical TBI severity and AUCs > 0.7 for radiographic TBI severity on D1 and W2. The relative utility of CRP and IL-6 for differentiating clinical and radiographic TBI severity also increased from D1 to W2, in contrast to most other biomarkers. Taken together, these results suggest IL-6, CRP, and SAA are particularly suitable for determining relative TBI severity when measured from the acute to subacute phase of injury, offering potential for establishing a role in assessing TBI severity within the domain of inflammatory biomarkers. This is consistent with prior studies that showed CRP is able to differentiate TBI with CT evidence of intracranial injury and is correlated with the injury severity score [29–31].

In our study, inflammatory marker levels were similar in GCS 13–15 TBI subjects and OCs (Tables 2 and 3) but were elevated in GCS 13–15 compared to HCs (Table 2). It is known that both GCS 13–15 TBI and orthopedic trauma induce systemic inflammation [32–36], and our findings suggest these two injury types induce similar levels of systemic inflammation when considering orthopedic trauma of AIS 1–3. While we cannot draw conclusions about their local patterns of inflammation, these findings provide impetus for closer examinations of precise levels of inflammatory biomarkers and their patterns of secretion to bridge the knowledge gaps between subgroups of TBI and persistent deficits in longitudinal outcomes.

Blood-based biomarkers are one of the four pillars of the 2025 NINDS “Clinical, Biomarker, Imaging, Modifier (CBI-M)” Framework for improving TBI characterization and classification [37]. The current acute CNS-specific biomarkers GFAP, UCH-L1, and S100B have shown promise in reducing radiation exposure associated with head CT scans in certain subpopulations and improving healthcare resource optimization in EDs for TBI patients who require different levels of care. However, their clinical use is currently limited to the first 12–24 h of injury. Our results suggest that incorporating such inflammatory biomarkers as IL-6, CRP, and SAA into the Biomarker Pillar of the CBI-M Framework warrants near-term examination. Given their ability to predict clinical and radiographic TBI severity at subacute timepoints, they may fulfill a unique role within contemporary advancements in the diagnostic framework for TBI.

### Subacute inflammatory biomarkers predict 6-month outcomes in TBI

Although acute (D1) inflammatory markers did not independently predict 6-month unfavorable functional outcomes in our study, several biomarkers measured in the subacute phase (W2) remained predictive of outcome after rigorous adjustment for known clinico-demographic predictors (Table 4). Importantly, the effect sizes of W2 SAA, CRP, IL-6, IL-15, and IL-17A were slightly attenuated but remained conserved after further adjusting for level of care (hospitalization status) at W2 (Table 5), supporting the consideration of subacute inflammatory biomarker signals as independent predictors of 6-month outcomes within the appropriate clinical context and without assigning full causality. Certainly, inflammatory cascades are initiated and modulated by distinct biomolecular signaling pathways from ongoing primary and secondary injuries and serve as quantitative measures of evolving comorbidity and injury burden at *a given point in time*. Taken together, our findings invite focused examinations of the mechanistic effects of specific biomarkers, biomarker clusters, and trajectories in hypothesis-driven research to elucidate their dynamic roles as indicators of injury progression and/or treatment response.

Our systematic assessments of 27 inflammatory biomarkers at D1 and W2 for their diagnostic and prognostic relevance at acute and subacute timepoints confirm and extend prior findings of association between smaller sets of biomarkers and functional outcomes [38–40] toward prospective clinical utility and implementation. Although 5 of these biomarkers were able to both differentiate TBI severity and predict 6-month outcomes, remaining biomarkers which were able to differentiate TBI severity (including IL-1ꞵ, IL-2, IL-4, IL-10, and TNF-α) or predict 6-month outcomes (including IL-5, MDC, MIP-1α, and TARC) deserve further exploration for diagnostic and prognostic utility, respectively.

Our findings distinguish inflammatory markers from structural TBI biomarkers, which predict unfavorable outcomes when drawn on D1 and decrease in circulating levels as they undergo systemic elimination [41]. In contrast, secondary inflammation is upregulated after traumatic injuries, and measurement of inflammatory markers may uniquely quantify their corresponding pathophysiologic processes after a complex injury such as TBI, with or without contributions from multisystem trauma. Inflammation is a “double-edged sword” in acute TBI [42]; it can exacerbate neuronal dysfunction, but can be beneficial for clearing damaged proteins, stimulating neurogenesis, and promoting recovery [43, 44]. Consistent with this hypothesis, prior evidence have reported on mixed relationships between elevated IL-6 levels with clinical outcomes when measured as peak levels shortly after acute TBI [40, 45–48], while persistently elevated IL-6 levels at 2-weeks correlate with unfavorable 6-month functional outcomes [17].

Chronic neuroinflammation after TBI is likely to represent a transformation from reactive inflammation to persistent pathology. Witcher et al. demonstrated that microglia-dependent inflammation in the subacute and chronic stages of TBI mediate behavioral and functional impairments [49], and this inflammation can be present for up to 17 years post-injury [50]. Similarly, markers of astrogliosis are elevated in patients with chronic symptoms after TBI and correlate with serum GFAP and total tau [51]. Persistent neuroinflammation has been proposed as a mechanism to link TBI and neurodegeneration [52–54]. Our finding that a subset of elevated subacute inflammatory biomarkers independently predicted worse functional outcomes advances the evidence on biomarker subtypes, their clinical utility, and potential for prospective implementation studies.

### Inflammatory cascades in TBI patients with impaired outcomes

In our study, elevated levels of inflammatory biomarkers at W2 were associated with unfavorable functional outcomes at 6-months. IL-15, IL-6, IL-17A, CRP, and SAA were among the most highly elevated biomarkers. Spearman correlations at W2 further showed that IL-6, IL-17A, CRP, and SAA show moderate-to-strong associations with each other (Fig. 2). As such, our findings suggest that patterns of acute phase reactants, IL-6, IL-17A, and IL-15 may predominate post-TBI inflammatory processes.

Persistent abnormal elevation of IL-6 stimulates a subsequent inflammatory cascade. IL-6 levels rise significantly after TBI in blood, cerebrospinal fluid (CSF), and brain tissue [55–57]. IL-6 is produced by neurons, astrocytes, and microglia in the CNS, as well as monocytes, macrophages, and B cells systemically [58]. It is both a marker of injury and mediator of inflammation, particularly when IL-6 elevation is high and/or persistent over time [59, 60]. IL-6 drives Th17 maintenance and thus IL-17 maintenance [61]. In children with mild TBI, IL-6 levels increase immediately after mild TBI and return to normal levels after 2-weeks. However, levels of IL-17 are expected to decrease immediately [62]. This downregulation of IL-17A after TBI occurs at the level of gene expression [63]. In our study, patients who did not experience the “return to normal” levels of IL-6 at W2 were more likely to have unfavorable 6-month outcomes. This is consistent with the findings of Kumar et al., which showed that patients with persistently elevated IL-6 in CSF experienced worse 6 month outcomes compared to those whose IL-6 levels decreased over time [17]. IL-17 is implicated in neuroinflammation in multiple sclerosis [64], neurodegenerative disorders [65], neuropsychiatric disorders, and secondary injury in TBI [66]. IL-6 also directly drives the synthesis of acute phase reactants, including CRP and SAA [67]. These markers have been proposed as biomarkers in TBI and may have, in part, a neuronal origin [30, 68–70], and may serve as chemoattractants and activators for inflammatory cells [69].

Of the inflammatory biomarkers in our study, IL-15 demonstrated a strong association with unfavorable outcome at 6-months. IL-15 is produced by astrocytes and microglia in the context of inflammatory CNS injury and induces CD8 + T cell and natural killer cell activation [71], leading to neuronal apoptosis in TBI and ischemic stroke [72, 73]. In our study, IL-15 was only moderately correlated with the levels of other inflammatory cytokines at W2, suggesting the pathophysiology behind its elevation may not be as tightly linked to the inflammatory cascade as previously postulated. Still, its role in regulating the inflammatory response to injury and neuronal apoptosis may have a significant role in mediating and propagating certain patterns of TBI outcomes [74].

### Potential of inflammatory biomarkers to inform therapeutic clinical trials

Since subacute elevations of inflammatory biomarkers can influence secondary injury and predict functional outcome, persistent inflammation after TBI may be a target for early therapeutic intervention. The majority of human trials to date on therapies for TBI, such as progesterone [75, 76], methylprednisolone [77], erythropoietin [78, 79], and minocycline [80, 81], have not shown definitive benefits for outcome, though these have been limited by largely dichotomous, single-point outcomes. Evidence from animal models on modulating chronic inflammation is mixed. Limiting chronic neuroinflammation after TBI by downregulating the chemokine receptor CX_3_CR1 prevented chronic degeneration and provided better functional recovery in a subset of mice [82]. Izzy et al. investigated an anti-CD3 monoclonal antibody in a mouse model of TBI, varying the timing of administration at immediate (4–6 h), early (3 days), or delayed (14 days). Immediate and early, but not delayed, delivery of this treatment resulted in improved behavioral outcomes [83]. The optimal timeframe for delayed administration of anti-inflammatory therapies is another potential avenue for future examinations.

### Limitations

We acknowledge limitations within this work. This study draws upon data from plasma levels of inflammatory biomarkers. Measuring biomarkers from the systemic circulation is sufficiently practical to implement in clinical care, but this is an indirect measure of inflammation in the CNS. In a systematic review and meta-analysis, Gigase et al. found poor correlation between inflammatory biomarkers in blood and CSF, though these studies did not focus specifically on TBI patients [84]. Despite this, evidence has demonstrated that blood markers of inflammation directly correlate with signs of injury in TBI [85–87], and work in animal models suggests that blood and CSF levels of IL-17A demonstrate similar trajectories after TBI [66].

We demonstrate that elevated inflammatory biomarkers in the subacute phase predict unfavorable long-term functional outcome, which was defined at 6-months post-injury. Unfortunately we were not able to determine whether TBI subjects had ongoing inflammatory conditions at subacute timepoints, such as pneumonia, nosocomial infections, and other systemic inflammatory processes, as complications data in the TRACK-TBI study were not recorded with time of onset, severity, duration, or resolution, and therefore were not appropriate for analysis. The strengths of association between candidate inflammatory biomarkers, ongoing medical and/or surgical comorbidities, and longitudinal outcomes would benefit from targeted and well-designed prospective studies. Similarly, the time courses of inflammatory cascades and their effects on longitudinal outcomes require further characterization, as we did not examine biomarker trajectories between D1 and W2, persistent biomarker elevations beyond W2, nor outcome measures prior to 6 months post-injury. Future studies integrating these timepoints of biomarker assessment will better identify their predictive capabilities at different timepoints of acute and subacute clinical care and better establish their role in the progression from acute to chronic inflammation. This may empower the design of therapeutic interventions to temporize inflammation after TBI and thereby improve outcomes.

We recognize that systemic inflammatory markers may be elevated in non-TBI acute and chronic inflammatory conditions (e.g., the acute stress response, autoimmune disorders, infection, malignancy, and others). Changes in biomarker levels as part of non-TBI systemic trauma should also be quantified and accounted for in future validation studies. We did not evaluate pre-existing inflammatory conditions or use of anti-inflammatory medications due to limited data availability on these factors. Important next steps include evaluating for endotypes amongst the most promising inflammatory markers, intracranial injury type and location, multi-dimensional outcomes, and changes in their diagnostic and prognostic ability when combined with CNS-specific biomarkers. As inflammatory biomarkers proceed to clinical implementation studies, definitive assessment of their interactions with biological factors such as sex remain of high importance [28]. Our exploratory finding of the interaction between 2-week TNF-α level and female sex had a wide confidence interval and was underpowered for prognostication. Hypothesis-driven studies with appropriate power calculations should be prioritized. The current study examined each biomarker separately. Advanced statistical modeling (e.g., dimension reduction) can identify clusters of markers with improved diagnostic or prognostic discriminability. These limitations await future prospective studies and near-term validation studies utilizing large, multicenter TBI consortium datasets with rigorous data standards, such as TRACK-TBI (https://tracktbi.ucsf.edu), CENTER-TBI (https://www.center-tbi.eu), and others.

## Conclusions

Ten circulating inflammatory proteins (CRP, SAA, IL-1ꞵ, IL-2, IL-4, IL-6, IL-10, IL-15, IL-17A, TNF-α) were associated with TBI diagnosis and severity at both D1 and W2 post-injury. Of these, five biomarkers (IL-15, IL-6, IL-17A, CRP, SAA) expressed subacute (W2) levels predictive of 6-month death/severe-disability, underscoring their near-term potential for validation as a novel class of diagnostic and predictive markers, and integration into existing and new prognostic models. Distillation of pro- and anti-inflammatory clusters and their mechanistic effects in acute brain trauma could facilitate precision medicine approaches for risk stratification and therapeutic modulation of TBI patients.

## Supplementary Information

Supplementary Material 1.

## Data Availability

Data from the TRACK-TBI study are available through the Federal Interagency Traumatic Brain Injury Research (FITBIR) Informatics System at https://doi.org/10.23718/FITBIR/1518881. Qualified researchers may request access to data stored in FITBIR which requires obtaining data access privileges outlined by FITBIR. TRACK-TBI study protocols and data collection forms are available at https://tracktbi.ucsf.edu/researchers. Investigators interested in investigating specific data elements may submit a Data Collaboration Request (https://tracktbi.ucsf.edu/collaboration-opportunities) to the TRACK-TBI Executive Committee. Statistical analyses of this study were supervised by Dr. Sonia Jain, PhD, Professor of Biostatistics at University of California, San Diego (San Diego, California, United States of America). Analytic code used to conduct the analyses in this study are not available in a public repository and may be made available upon email request to the corresponding author. TRACK-TBI study protocols, informed consent forms, data collection forms, and data dictionaries are available for public access at https://tracktbi.ucsf.edu/researchers.

## Abbreviations

AIS: Abbreviated Injury Scale
AOR: Adjusted odds ratio
AUC: Area under the curve
CDE: Common Data Element
CI: Confidence interval
CNS: Central nervous system
CONSORT: Consolidated Standards of Reporting Trials
CRP: C-reactive protein
CT: Computed tomography
CV: Coefficient of variation
D1: Day 1
ED: Emergency department
FDA: Food and Drug Administration
FITBIR: Federal Interagency Traumatic Brain Injury Research
GCS: Glasgow Coma Scale
GFAP: Glial fibrillary acidic protein
GOSE: Glasgow Outcome Scale-Extended
HC: Healthy control
IFN: Interferon
IL: Interleukin
IP: Interferon-gamma induced protein
IRB: Institutional review board
K2EDTA: Dipotassium ethylenediaminetetraacetic acid
LLOQ: Lower limit of quantification
MCP: Monocyte chemoattractant protein
MDC: Macrophage-derived chemokine
MIP: Macrophage inflammatory protein
MSD: Meso Scale Discovery
NINDS: National Institute of Neurological Disorders and Stroke
OC: Orthopedic trauma control
OR: Odds ratio
ROC: Receiver-operating characteristic
S100B: S100 calcium-binding protein B
SAA: Serum amyloid A
TARC: Thymus and activation regulated chemokine
TBI: Traumatic brain injury
TNF: Tumor necrosis factor
TRACK-TBI: Transforming Research and Clinical Knowledge in Traumatic Brain Injury
UCH-L1: Ubiquitin c-terminal hydrolase-L1
ULOQ: Upper limit of quantification
USA: United States of America
W2: 2-Week
