# Supplementary material for: Inflammatory blood-based biomarkers to aid in the assessment and prognostication of traumatic brain injury: a TRACK-TBI study
Source: J Neuroinflammation. 2026 May 28;23:261. doi: 10.1186/s12974-026-03891-3 (PMC13430862; doi:10.1186/s12974-026-03891-3)
Supplement: Supplementary file 1 — Supplementary Material 1. [file 12974_2026_3891_MOESM1_ESM.docx]

**SUPPLEMENTARY MATERIAL**

**Supplementary Table S1.** Assay Characteristics, Sensitivity, and Precision

| **Biomarker** | **Intra-assay %CV** | **Inter-assay %CV** | **Dynamic Range (LLOQ – ULOQ)** |
| --- | --- | --- | --- |
| CRP | 7.5 | 6.9 | 1.33 - 49600 pg/mL |
| SAA | 5.9 | 7.5 | 10.9 - 138000 pg/mL |
| Eotaxin | 8.6 | 9.7 | 3.26 - 1120 pg/mL |
| Eotaxin-3 | 1.7 | 5.7 | 1.77 - 3750 pg/mL |
| IFN-γ | 7.6 | 9.7 | 0.37 - 938 pg/mL |
| IL-1α | 3.5 | 3.9 | 0.09 - 278 pg/mL |
| IL-1β | 1.7 | 4.8 | 0.05 - 375 pg/mL |
| IL-2 | 14.9 | 13.0 | 0.09 - 938 pg/mL |
| IL-4 | 11.5 | 20.0 | 0.02 - 158 pg/mL |
| IL-5 | 2.5 | 9.2 | 0.14 - 562 pg/mL |
| IL-6 | 10.0 | 14.5 | 0.06 - 488 pg/mL |
| IL-7 | 17.0 | 15.1 | 0.12 - 563 pg/mL |
| IL-10 | 19.0 | 21.5 | 0.04 - 233 pg/mL |
| IL-12/23p40 | 12.9 | 18.3 | 0.33 - 2250 pg/mL |
| IL-12p70 | 17.6 | 22.3 | 0.11 - 315 pg/mL |
| IL-15 | 6.0 | 9.4 | 0.15 - 525 pg/mL |
| IL-16 | 1.7 | 3.9 | 2.83 - 1870 pg/mL |
| IL-17A | 11.0 | 11.2 | 0.31 - 3650 pg/mL |
| IP-10 | 8.7 | 12.3 | 0.37 - 500 pg/mL |
| MCP-1 | 3.2 | 7.9 | 0.09 - 375 pg/mL |
| MCP-4 | 14.0 | 21.7 | 0.18 - 472 pg/mL |
| MDC | 1.8 | 9.7 | 1.22 - 3700 pg/mL |
| MIP-1α | 4.2 | 9.2 | 3.02 - 743 pg/mL |
| MIP-1β | 6.6 | 18.7 | 0.17 - 520 pg/mL |
| TARC | 11.5 | 11.2 | 0.22 - 1120 pg/mL |
| TNF-α | 10.1 | 11.4 | 0.04 - 248 pg/mL |
| TNF-β | 8.7 | 10.9 | 0.08 - 458 pg/mL |

**Caption:** Assay characteristics for each inflammatory proteomic biomarker are presented, including intra- and inter-assay coefficients of variation (%CV) and the dynamic range from the lower limit of quantification (LLOQ) to the upper limit of quantification (ULOQ). CRP = c-reactive protein; IFN-γ = interferon γ; IL = interleukin; IL-12/IL-23p40 = IL-12/IL-23 p40 protein; IL-12 p70 = IL-12 p70 protein; IP-10 = interferon gamma-induced protein 10; MCP = monocyte chemoattractant protein; MDC = macrophage-derived chemokine; MIP = macrophage inflammatory protein; SAA = serum amyloid A; TARC = thymus- and activation-regulated chemokine; TNF = tumor necrosis factor

**Supplementary Table S2.** Logistic Regression Models for Subacute (2-Week) Inflammatory Biomarkers, Female Sex, and Biomarker-Sex Interaction as Predictors of 6-Month Unfavorable Outcome (GOSE 1-4 vs. 5-8)

|  | **2-Week Biomarker as Predictor** | |
| --- | --- | --- |
| **Biomarker** | **AOR (95% CI)** | **p-value** |
| CRP | **4.09 (1.90-8.81)** | **<0.001** |
| Sex | 0.21 (0.03-1.82) | 0.157 |
| Interaction | 4.03 (0.60-27.20) | 0.152 |
| SAA | **8.92 (3.53-22.56)** | **<0.001** |
| Sex | 0.88 (0.16-4.91) | 0.886 |
| Interaction | 1.10 (0.26-4.64) | 0.896 |
| IL-1β | 1.23 (0.82-1.86) | 0.322 |
| Sex | 0.74 (0.29-1.89) | 0.528 |
| Interaction | 1.61 (0.63-4.07) | 0.318 |
| IL-2 | 1.45 (0.91-2.31) | 0.115 |
| Sex | 0.62 (0.21-1.83) | 0.390 |
| Interaction | 1.44 (0.57-3.62) | 0.443 |
| IL-4 | 1.11 (0.76-1.63) | 0.581 |
| Sex | 0.68 (0.26-1.79) | 0.438 |
| Interaction | 1.24 (0.48-3.21) | 0.657 |
| IL-6 | **3.83 (1.92-7.64)** | **<0.001** |
| Sex | 0.39 (0.07-2.15) | 0.282 |
| Interaction | 2.63 (0.56-12.39) | 0.221 |
| IL-10 | **1.69 (1.04-2.74)** | **0.034** |
| Sex | 0.72 (0.26-1.99) | 0.531 |
| Interaction | 0.91 (0.44-1.86) | 0.786 |
| IL-15 | **2.01 (1.28-3.16)** | **0.003** |
| Sex | 0.75 (0.26-2.12) | 0.580 |
| Interaction | 1.62 (0.58-4.52) | 0.361 |
| IL-17A | **2.43 (1.47-4.02)** | **0.001** |
| Sex | 0.33 (0.08-1.44) | 0.140 |
| Interaction | 2.88 (0.76-10.93) | 0.121 |
| TNF-α | 1.47 (0.92-2.35) | 0.112 |
| Sex | 0.23 (0.05-1.15) | 0.073 |
| Interaction | **10.49 (1.59-68.97)** | **0.014** |

**Caption:** Multivariable logistic regression results for 6-month unfavorable outcomes (GOSE 1-4 vs. 5-8) are shown for 2-week biomarker, sex, and the biomarker-sex interaction factor. For biomarkers, adjusted odds ratios (AOR) and their 95% confidence intervals (CI) represent the change in odds per log_2_-unit increase in biomarker level. Models were fit separately for each biomarker and adjusted for age (per-year), sex (male/female), major extracranial injury (AIS ≥3 for extracranial body systems; yes/no), emergency department arrival GCS (3-12 vs. 13-15), psychiatric history (yes/no), Marshall CT score (1 vs. 2, 3-4, 5-6) and log_2_-transformed day 1 GFAP levels (per-unit). P-values of nominal significance (p<0.05) are bolded. P-values were not corrected for multiple comparisons for this exploratory sensitivity analysis. AIS = Abbreviated Injury Scale; CRP = C-reactive protein; CT = computed tomography; GCS = Glasgow Coma Scale; GFAP = glial fibrillary acidic protein; GOSE = Glasgow Outcome Scale-Extended; IL = interleukin; SAA = serum amyloid A; TNF = tumor necrosis factor

**Supplementary Table S3.** Acute Care Length of Stay, 2-Week Hospitalization Status, and 6-Month Outcome Category

| **Variable** | **GOSE 1-4**  **N (%)** | **GOSE 5-8**  **N (%)** | **p-value** |
| --- | --- | --- | --- |
| Hospital LOS |  |  | <0.0001 |
| 0-7 days | 5 (7.1) | 222 (74.3) |  |
| 7-14 days | 3 (4.3) | 28 (9.4) |  |
| >14 days | 62 (88.6) | 49 (16.4) |  |
| ICU LOS |  |  | <0.0001 |
| 0-7 days | 20 (28.6) | 251 (83.9) |  |
| 7-14 days | 7 (10.0) | 23 (7.7) |  |
| >14 days | 43 (61.4) | 25 (8.4) |  |
| Hospitalization status at 2-weeks post-injury |  |  | <0.0001 |
| Not hospitalized | 8 (11.4) | 250 (83.6) |  |
| Hospitalized, not in ICU | 19 (27.1) | 24 (8.0) |  |
| Hospitalized, in ICU | 43 (61.4) | 25 (8.4) |  |

**Caption:** Hospital LOS, ICU LOS, and hospitalization status at 2-weeks post-injury in TBI patients with 6-month unfavorable (GOSE 1-4) vs. favorable (GOSE 5-8) functional outcomes. Parentheses represent percentages. GOSE = Glasgow Outcome Scale-Extended; ICU = intensive care unit; LOS = length of stay

**Supplementary Table S4.** Demographic and Clinical Characteristics of TBI Subjects By 6-Month Outcome Category

| **Variable** | **Total**  **N (%)** | **GOSE 1-4**  **N (%)** | **GOSE 5-8**  **N (%)** | **p-value** | **GOSE 1-7**  **N (%)** | **GOSE 8**  **N (%)** | **p-value** |
| --- | --- | --- | --- | --- | --- | --- | --- |
| **Age** (Median [Q1-Q3]) | 38 [26-52] | 37 [25-57] | 38 [26-51] | 0.451 | 38 [26-53] | 36 [27-49] | 0.733 |
| **Log_2_ GFAP** (Median [Q1-Q3]) | 10.36 [7.85-11.47] | 11.47 [11.42-11.47] | 9.75 [7.26-11.47] | <0.001 | 10.81 [7.97-11.47] | 9.69 [7.56-11.01] | 0.005 |
| **Sex** |  |  |  | 0.243 |  |  | 0.075 |
| Male | 261 (70.7) | 54 (77.1) | 207 (69.2) |  | 180 (67.9) | 81 (77.9) |  |
| Female | 108 (29.3) | 16 (22.9) | 92 (30.8) |  | 85 (32.1) | 23 (22.1) |  |
| Total | 369 (100) | 70 (100) | 299 (100) |  | 265 (100) | 104 (100) |  |
| **Race** |  |  |  | 0.941 |  |  | 0.530 |
| White | 286 (77.7) | 55 (79.7) | 231 (77.3) |  | 206 (78.0) | 80 (76.9) |  |
| Black | 55 (15.0) | 9 (13.0) | 46 (15.4) |  | 41 (15.5) | 14 (13.5) |  |
| Other | 27 (7.3) | 5 (7.3) | 22 (7.3) |  | 17 (6.4) | 10 (9.6) |  |
| Total | 368 (100) | 69 (100) | 299 (100) |  | 264 (100) | 104 (100) |  |
| **Hispanic** |  |  |  | 0.081 |  |  | 0.444 |
| Non-Hispanic | 305 (82.9) | 53 (75.7) | 252 (84.6) |  | 216 (81.8) | 89 (85.6) |  |
| Hispanic | 63 (17.1) | 17 (24.3) | 46 (15.4) |  | 48 (18.2) | 15 (14.4) |  |
| Total | 368 (100) | 70 (100) | 298 (100) |  | 264 (100) | 104 (100) |  |
| **Psychiatric History** |  |  |  | 0.350 |  |  | 0.006 |
| No | 284 (77.0) | 57 (81.4) | 227 (75.9) |  | 194 (73.2) | 90 (86.5) |  |
| Yes | 85 (23.0) | 13 (18.6) | 72 (24.1) |  | 71 (26.8) | 14 (13.5) |  |
| Total | 369 (100) | 70 (100) | 299 (100) |  | 265 (100) | 104 (100) |  |
| **Prior TBI** |  |  |  | 0.863 |  |  | 0.769 |
| No | 265 (78.2) | 48 (80.0) | 217 (77.8) |  | 190 (77.6) | 75 (79.8) |  |
| Yes | 74 (21.8) | 12 (20.0) | 62 (22.2) |  | 55 (22.4) | 19 (20.2) |  |
| Total | 339 (100) | 60 (100) | 279 (100) |  | 245 (100) | 94 (100) |  |
| **Injury Cause** |  |  |  | 0.869 |  |  | 0.889 |
| Road traffic incident | 231 (62.8) | 43 (61.4) | 188 (63.1) |  | 167 (63.0) | 64 (62.1) |  |
| Incidental fall | 83 (22.6) | 15 (21.4) | 68 (22.8) |  | 60 (22.6) | 23 (22.3) |  |
| Violence/assault | 21 (5.6) | 5 (7.2) | 16 (5.4) |  | 16 (6.0) | 5 (4.9) |  |
| Other | 33 (9.0) | 7 (10.0) | 26 (8.7) |  | 22 (8.3) | 11 (10.7) |  |
| Total | 368 (100) | 70 (100) | 298 (100) |  | 265 (100) | 103 (100) |  |
| **ED Disposition** |  |  |  | <0.001 |  |  | <0.001 |
| ED Discharge | 63 (17.1) | 1 (1.4) | 62 (20.7) |  | 34 (12.8) | 29 (27.9) |  |
| Hospital Ward | 86 (23.3) | 2 (2.9) | 84 (28.1) |  | 55 (20.8) | 31 (29.8) |  |
| Intensive Care Unit | 220 (59.6) | 67 (95.7) | 153 (51.2) |  | 176 (66.4) | 44 (42.3) |  |
| Total | 369 (100) | 70 (100) | 299 (100) |  | 265 (100) | 104 (100) |  |
| **Major Extracranial Injury** |  |  |  | 0.031 |  |  | 0.008 |
| No | 277 (75.1) | 45 (64.3) | 232 (77.6) |  | 189 (71.3) | 88 (84.6) |  |
| Yes | 92 (24.9) | 25 (35.7) | 67 (22.4) |  | 76 (28.7) | 16 (15.4) |  |
| Total | 369 (100) | 70 (100) | 299 (100) |  | 265 (100) | 104 (100) |  |
| **Loss of Consciousness** |  |  |  | 0.093 |  |  | 0.536 |
| No | 34 (9.2) | 2 (2.9) | 32 (10.7) |  | 22 (8.3) | 12 (11.5) |  |
| Yes | 322 (87.3) | 66 (94.3) | 256 (85.6) |  | 234 (88.3) | 88 (84.6) |  |
| Unknown | 13 (3.5) | 2 (2.9) | 11 (3.7) |  | 9 (3.4) | 4 (3.9) |  |
| Total | 369 (100) | 70 (100) | 299 (100) |  | 265 (100) | 104 (100) |  |
| **Post-Traumatic Amnesia** |  |  |  | 0.060 |  |  | 0.027 |
| No | 31 (8.4) | 5 (7.1) | 26 (8.7) |  | 19 (7.2) | 12 (11.5) |  |
| Yes | 266 (72.1) | 44 (62.9) | 222 (74.3) |  | 186 (70.2) | 80 (76.9) |  |
| Unknown | 72 (19.5) | 21 (30) | 51 (17.1) |  | 60 (22.6) | 12 (11.5) |  |
| Total | 369 (100) | 70 (100) | 299 (100) |  | 265 (100) | 104 (100) |  |
| **Intracranial Injury on CT** |  |  |  | <0.001 |  |  | <0.001 |
| CT- | 148 (40.1) | 3 (4.3) | 145 (48.5) |  | 87 (32.8) | 61 (58.7) |  |
| CT+ | 221 (59.9) | 67 (95.77) | 154 (51.5) |  | 178 (67.2) | 43 (41.4) |  |
| Total | 369 (100) | 70 (100) | 299 (100) |  | 265 (100) | 104 (100) |  |
| **Marshall CT Score** |  |  |  | <0.001 |  |  | <0.001 |
| 1 | 148 (40.6) | 3 (4.3) | 145 (49.2) |  | 87 (33.2) | 61 (59.2) |  |
| 2 | 134 (36.7) | 22 (31.4) | 112 (38.0) |  | 98 (37.4) | 36 (35.0) |  |
| 3-4 | 18 (4.9) | 8 (11.4) | 10 (3.4) |  | 17 (6.5) | 1 (1.0) |  |
| 5-6 | 65 (17.8) | 37 (52.9) | 28 (9.5) |  | 60 (22.9) | 5 (4.9) |  |
| Total | 365 (100) | 70 (100) | 295 (100) |  | 262 (100) | 103 (100) |  |

**Caption:** Demographics and clinical characteristics of TBI subjects with incomplete functional recovery (GOSE 1-7) vs. complete functional recovery (GOSE 8) at 6-months post-injury. Major extracranial injury represented injury to a body system below the head and neck with an Abbreviated Injury Scale score ≥3. P-values were not corrected for multiple comparisons. CT = computed tomography; ED = emergency department; GCS = Glasgow Coma Scale; GFAP = glial fibrillary acidic protein; GOSE = Glasgow Outcome Scale - Extended; ICU = intensive care unit; Q1-Q3 = quartile 1 to quartile 3; TBI = traumatic brain injury

**Supplementary Table S5.** Logistic Regression Models for Acute (Day 1) and Subacute (2-Week) Inflammatory Biomarkers as Predictors of 6-Month Incomplete Recovery (GOSE 1-7 vs. 8)

|  | **Day 1 Biomarker as Predictor** | | | | **2-Week Biomarker as Predictor** | | | |
| --- | --- | --- | --- | --- | --- | --- | --- | --- |
| **Biomarker** | **Raw OR**  **(95% CI)** | **AOR**  **(95% CI)** | **Uncorrected p-value** | **Corrected p-value** | **Raw OR (95% CI)** | **AOR**  **(95% CI)** | **Uncorrected p-value** | **Corrected p-value** |
| CRP | 1.10  (1.01-1.21) | 0.93  (0.81-1.07) | 0.314 | 0.886 | 1.18  (1.08-1.28) | 0.94  (0.81-1.07) | 0.342 | 0.513 |
| SAA | 1.11  (1.01-1.22) | 1.03  (0.88-1.2) | 0.709 | 0.954 | 1.24  (1.13-1.37) | 1.07  (0.92-1.23) | 0.386 | 0.549 |
| Eotaxin | 0.84  (0.62-1.14) | 0.95  (0.63-1.43) | 0.799 | 0.954 | 1.04  (0.73-1.47) | 0.85  (0.53-1.37) | 0.516 | 0.606 |
| Eotaxin-3 | 0.82  (0.66-1.02) | 0.89  (0.69-1.16) | 0.396 | 0.891 | 0.91  (0.73-1.13) | 0.84  (0.64,-1.09) | 0.194 | 0.436 |
| IFN-γ | 1.02  (0.85-1.22) | 1.06  (0.84-1.32) | 0.636 | 0.954 | 0.88  (0.74-1.05) | 0.79  (0.63-0.99) | 0.040 | 0.331 |
| IL-1α | 1.17  (0.94-1.45) | 1.25  (0.97-1.62) | 0.081 | 0.641 | 1.15  (0.91-1.45) | 1.19  (0.90-1.56) | 0.220 | 0.457 |
| IL-1β | 1.24  (1.07-1.45) | 0.95  (0.77-1.18) | 0.638 | 0.954 | 1.12  (0.92-1.37) | 0.82  (0.62-1.07) | 0.145 | 0.434 |
| IL-2 | 1.40  (1.20-1.64) | 1.14  (0.90-1.43) | 0.276 | 0.886 | 1.20  (0.99-1.44) | 0.76  (0.59-1.00) | 0.049 | 0.331 |
| IL-4 | 1.31  (1.12-1.53) | 1.04  (0.86-1.26) | 0.681 | 0.954 | 1.20  (0.95-1.51) | 0.81  (0.59-1.10) | 0.177 | 0.434 |
| IL-5 | 1.17  (0.86-1.59) | 1.01  (0.69-1.49) | 0.954 | 0.954 | 1.09  (0.88-1.35) | 0.80  (0.59-1.07) | 0.131 | 0.434 |
| IL-6 | 1.29  (1.12-1.49) | 1.22  (0.97-1.54) | 0.095 | 0.641 | 1.33  (1.17-1.52) | 0.95  (0.77-1.17) | 0.617 | 0.686 |
| IL-7 | 0.70  (0.56-0.88) | 0.76  (0.59-0.99) | 0.044 | 0.641 | 1.02  (0.85-1.22) | 0.79  (0.62-0.99) | 0.042 | 0.331 |
| IL-10 | 1.32  (1.16-1.50) | 1.02  (0.84-1.24) | 0.845 | 0.954 | 1.39  (1.16-1.68) | 1.01  (0.80-1.29) | 0.906 | 0.906 |
| IL-12/23p40 | 0.91  (0.72-1.15) | 0.90  (0.68-1.19) | 0.466 | 0.954 | 0.85  (0.64-1.11) | 0.78  (0.57-1.09) | 0.144 | 0.434 |
| IL-12p70 | 1.32  (1.07-1.63) | 1.05  (0.81-1.37) | 0.688 | 0.954 | 1.27  (1.03-1.57) | 0.94  (0.73-1.23) | 0.664 | 0.690 |
| IL-15 | 1.78  (1.23-2.58) | 0.92  (0.52-1.64) | 0.780 | 0.954 | 1.79  (1.18-2.72) | 0.73  (0.43-1.25) | 0.251 | 0.466 |
| IL-16 | 1.19  (0.97-1.46) | 0.87  (0.67-1.15) | 0.328 | 0.886 | 0.89  (0.70-1.11) | 0.75  (0.55-1.03) | 0.074 | 0.333 |
| IL-17A | 1.43  (1.20-1.71) | 1.21  (0.97-1.50) | 0.088 | 0.641 | 1.33  (1.14-1.55) | 1.05  (0.86-1.28) | 0.635 | 0.686 |
| IP-10 | 1.06  (0.84-1.34) | 1.01  (0.72-1.42) | 0.943 | 0.954 | 1.20  (0.89-1.63) | 0.81  (0.54-1.20) | 0.291 | 0.491 |
| MCP-1 | 1.16  (0.94-1.42) | 0.90  (0.65-1.23) | 0.499 | 0.954 | 1.02  (0.72-1.45) | 0.64  (0.40-1.03) | 0.067 | 0.333 |
| MCP-4 | 0.63  (0.50-0.80) | 0.81  (0.60-1.09) | 0.166 | 0.886 | 1.03  (0.81-1.33) | 0.89  (0.65-1.22) | 0.458 | 0.589 |
| MDC | 1.00  (0.68-1.48) | 1.03  (0.57-1.87) | 0.915 | 0.954 | 0.67  (0.44-1.01) | 0.83  (0.48-1.43) | 0.496 | 0.606 |
| MIP-1α | 0.99  (0.70-1.40) | 0.83  (0.54-1.27) | 0.382 | 0.891 | 1.06  (0.76-1.48) | 0.79  (0.53-1.19) | 0.259 | 0.466 |
| MIP-1β | 0.88  (0.68-1.15) | 0.81  (0.58-1.13) | 0.215 | 0.886 | 0.82  (0.61-1.11) | 0.63  (0.42-0.94) | 0.023 | 0.331 |
| TARC | 0.76  (0.62-0.92) | 0.88  (0.71-1.09) | 0.250 | 0.886 | 0.87  (0.73-1.04) | 0.92  (0.74-1.14) | 0.429 | 0.579 |
| TNF-α | 1.47  (1.20-1.80) | 1.01  (0.75-1.36) | 0.925 | 0.954 | 1.35  (1.01-1.81) | 0.76  (0.50-1.13) | 0.176 | 0.434 |
| TNF-β | 1.03  (0.54-1.95) | 0.89  (0.43-1.82) | 0.748 | 0.954 | 0.54  (0.30-0.98) | 0.71  (0.36-1.43) | 0.338 | 0.513 |

**Caption:** Univariate and multivariable logistic regression models for incomplete vs. complete 6-month functional recovery (GOSE 1-7 vs. 8) are shown for plasma biomarkers measured on day 1 and at 2-weeks. Unadjusted odds ratios (OR), adjusted odds ratios (AOR), and 95% confidence intervals (CI) represent the change in odds per log_2_-unit increase in biomarker level. Multivariable models were fit separately for each biomarker and adjusted for age (per-year), sex (male/female), major extracranial injury (AIS ≥3 for extracranial body systems; yes/no), emergency department arrival GCS (3-12 vs. 13-15), psychiatric history (yes/no), Marshall CT score (1 vs. 2, 3-4, 5-6), and log_2_-transformed day 1 GFAP levels. Uncorrected p-values are shown for multivariable models, and were corrected for multiple comparisons using the Benjamini-Hochberg method. AIS = Abbreviated Injury Scale; CRP = C-reactive protein; CT = computed tomography; GFAP = glial fibrillary acidic protein; GOSE = Glasgow Outcome Scale-Extended; IFN = interferon; IL = interleukin; IP = interferon-gamma induced protein; MCP = monocyte chemoattractant protein; MDC = macrophage-derived chemokine; MIP = macrophage inflammatory protein; SAA = serum amyloid A; TARC = thymus and activation regulated chemokine; TNF = tumor necrosis factor
